# A posteriori error approximation in discontinuous Galerkin method on polygonal meshes in elliptic problems

**DOI:** 10.1038/s41598-023-37414-4

**Published:** 2023-07-04

**Authors:** Jan Jaśkowiec, Jerzy Pamin

**Affiliations:** Chair for Computational Engineering, Faculty of Civil Engineering, Cracow University of Technology, Warszawska 24, 31-155 Cracow, Poland

**Keywords:** Civil engineering, Mechanical engineering, Applied mathematics, Computational science

## Abstract

The paper presents a posteriori error approximation concept based on residuals in the two-dimensional discontinuous Galerkin (DG) method. The approach is relatively simple and effective in application, and it takes advantage of some unique properties of the DG method. The error function is constructed in an enriched approximation space, utilizing the hierarchical nature of the basis functions. Among many versions of the DG method, the most popular one is based on the interior penalty approach. However, in this paper a DG method with finite difference (DGFD) is utilized, where the continuity of the approximate solution is enforced by finite difference conditions applied on the mesh skeleton. In the DG methods arbitrarily shaped finite elements can be used, so in this paper the meshes with polygonal finite elements are considered, including quadrilateral and triangular elements. Some benchmark examples are presented, in which Poisson’s and linear elasticity problems are considered. The examples use various mesh densities and approximation orders to evaluate the errors. The error estimation maps, generated for the discussed tests, indicate a good correlation with the exact errors. In the last example, the error approximation concept is applied for an adaptive *hp* mesh refinement.

## Introduction

This paper presents an error approximation concept for a version of the discontinuous Galerkin (DG) method called the discontinuous Galerkin with finite difference (DGFD) method, introduced in the first author’s previous papers^1–5^. The main characteristic of the DGFD method is a stabilization parameter $w$, interpreted as a small distance from the mesh skeleton. In the DGFD method, like in any other version of the DG method, arbitrary global approximation can be constructed on the entire domain with arbitrary basis functions in elements. In this paper Chebyshev polynomials are employed as the approximation basis since their construction does not require nodes and, as such, they do not depend on the shape of finite elements. In consequence, arbitrary polygonal finite elements can be used in the mesh. The Chebyshev polynomials can be constructed in a recursive manner, which helps one to avoid significant truncation errors when constructing higher-order polynomials. These basis function properties are utilized for error approximation.

Error estimation is a common problem in all computational methods. It is used to assess the quality of the approximate solution. When it is applied for mesh refinement, error estimation makes it possible to obtain a better solution with minimum computational costs. In any automatic mesh adaptation, the procedure of error estimation is the most important component. Therefore, this paper is focused on error estimation techniques in the DGFD method to be applied in an automatic *hp* mesh refinement.

The DG method has a long history that goes back to the early seventies of the previous century^6^. A constant development has been observed since that time^7–9^. In general, the DG method can be treated as a particular version of the finite element method. In the DG method the domain is discretized using a finite element mesh, and the basis functions are constructed within the finite elements. The main difference between those two methods is the construction of the global approximation space. In the standard FEM, the global approximation space is continuous a-priori, while in the DG method the global approximation space is not continuous, so some techniques have to be employed to make the final approximate solution continuous. The lack of such continuity in the DG method seems to be its drawback, but on the other hand, it provides great flexibility in applying the basis functions and the shapes of the finite elements. It is natural for the DG methods to use polygonal/polyhedral finite elements. Moreover, the orders of approximation in the finite elements can be selected independently for each element. It enables an efficient *hp*-adaptation procedure where the finite element division or modification of the approximation order can be performed in a single element without any problem. In contrast, the *hp*-adaptation in the FEM affects the neighboring elements. In general, the DG method can be seen as a particular version of FEM, which gives new opportunities in the numerical analysis, but we have to deal with the discontinuities.

Since in the DG methods, unlike in the FEM, the approximation functions passing from one finite element to another one are not continuous, integration along the mesh skeleton (inter-element borders) is needed. Depending on the version of the DG method used, the continuity of the final solution is enforced by different conditions related to the mesh skeleton. In the interior penalty DG (IPDG) method, the most popular in literature^10–12^, such continuity is obtained with the use of the Nitsche method^13^ which utilizes numerical fluxes applied to the skeleton and an additional penalty-like term, called stabilization parameter. In the local DG (LDG) methods^14–16^, the Bassi-Rebay DG^17^ or the Baumann-Oden DG^18^, two numerical fluxes are used for the primary and secondary fields. This is due to the ultra-weak problem formulation, in which a second-order problem is written with two first-order equations. These numerical fluxes utilize additional parameters that must be evaluated. The ultra-weak formulation is also applied in the hybridized DG (HDG) method^19,20^. Hybridization is the process of removing continuity constraints of finite element spaces without altering the solution. The number of unknowns in this method is strongly reduced by the hybridization. The HDG method has been applied to various engineering problems like second order elliptic problems^21^, incompressible Navier–Stokes equations^22^, space-time fractional advection-dispersion equations^23^, or the flow in petroleum reservoirs^24^ . An alternative approach is adopted in the discontinuous Petrov-Galerkin (DGP) method^25,26^, or the DG method with Lagrange multipliers (DGLM)^27^, where the numerical fluxes are treated as additional unknowns. Extensive overviews of some of the DG methods are presented in.douglassps2002,Kirby2005.

Error estimation has been discussed in a number of scientific articles referring to various computational methods, for instance the finite element^30–33^ or meshless methods^34,35^. Recently, the a posteriori error estimation and mesh adaptation in the DG methods have gained increasing attention. Some techniques for error estimation in hyperbolic or elliptic problems have also been proposed. The error can be estimated using a dual problem formulation^36^, where orthogonal polynomials, defined on triangular elements^37^, are used to evaluate the error in each element. The modified version of the DG method exhibits superconvergence properties that can be adopted for error estimation^38,39^. Radau polynomials have been applied in a local steady state problem in each element to approximate the error^40^. In another DG application an enriched polynomial basis is utilized in each tetrahedral element for error estimation^41^. A global estimator in the $L_2$ norm is proposed^42^ for elliptic problems. A residual-based error estimator and a material force concept have been used to estimate the error on quadtree meshes^43^. A posteriori error estimation in terms of the energy norm has also been considered in the interior penalty DG method to solve an elliptic boundary-value problem^44^. An eigenvalue problem has been analyzed^45^ with the DG method, in the context of an auxiliary subspace technique applied for error estimation^46^. Special attention should be paid to the works of M. Baccouch concerning error estimation in the DG methods, as they have made a significant contribution to computational analysis using the DG methods^47–61^. His articles focus mainly on the LDG methods for one-dimensional or two-dimensional domains discretised with Cartesian grids. Another approach is proposed in^62^ where the error in the elements is based on the oscillations in the right-hand side function when projected to the approximation space.

In this paper an original technique to approximate the error in the DGFD method is proposed. In this method, the values of the trial function gradients on the mesh skeleton and on the Dirichlet boundary are approximated using finite difference formulas. In consequence, the non-zero values in the residuum functions have their source in the difference between the approximated values on the mesh skeleton and the ones obtained in the approximate solution. The error function can be constructed in the same function space as the approximate solution, but to obtain a better error approximation an enriched space is recommended. The approximation in the finite elements is based on the Chebyshev hierarchical polynomials. The element stiffness matrix also has a hierarchical structure. It enables the *p* upgrade of the approximation in the finite elements and in consequence the upgraded global stiffness matrix can be constructed and factorized in an effective way. This property is utilized in the error approximation solver, which can be obtained with a minimum effort.

The error evaluation method presented in this paper is illustrated by some benchmark examples, where the exact error function is compared with the approximate one. In these examples demanding problems are analyzed where the exact solutions have localized large gradients. Moreover, various types of meshes are considered with different approximation orders in the elements. Each time the error is approximated with a high accuracy. In the last example, an *hp* refinement is performed based on the approximated error.

This paper is focused on two-dimensional (2D) boundary value problems. However, in the authors’ opinion, the proposed method of error approximation can be directly applied to three-dimensional (3D) problems with polyhedra meshes. In this paper, the error approximation concept is applied to two types of continuous elliptic problems: Poisson’s problem and linear elasticity. As a direct continuation of the research, the methods of error approximation can be applied to other types of problems, where the exact solutions have discontinuous fields, for example, for domains with cracks or hyperbolic partial differential equations.

This paper is organized as follows. In  “Problem formulation” Section two elliptic problems are defined and analyzed, namely the Poisson’s and elasticity problem. Their weak forms are derived for the DGFD method in “Weak formulations” Section. Some questions concerning the approximation in the DGFD method are discussed in “Approximation in DG method” Section. The stability parameter plays a crucial role in the error approximation, so the discussion on its influence on the approximate solution is presented in “Discussion on the stability parameter” Section. “Error estimation method” Section presents the proposed method of error approximation, which is subsequently illustrated by four examples provided in ’Examples” Section. The paper ends with brief conclusions.

## Problem formulation

This paper utilizes two types of two-dimensional linear elliptic boundary value problems to present a new error approximation method. The problems are defined on domain $\varOmega$ with outer boundary ${\varGamma }$. The first one is the scalar Poisson’s problem with the following boundary conditions

1 $$\begin{aligned} -&\Delta u= f\qquad \text {in } \varOmega \\ {}&u= {\hat{u}} \quad {\text{on } } {\varGamma }{^{D}}\,,\quad {\varvec{\nabla }}u\cdot \textbf{n}= {\hat{h}} \qquad {\text{on }} {\varGamma }{^{N}} \end{aligned}$$

where ${\varGamma }^D$ and ${\varGamma }^{N}$ are the parts of the outer boundary with Dirichlet and Neumann boundary conditions, respectively, and ${\varGamma }^D\cup {\varGamma }^{N} = {\varGamma }$, ${\varGamma }^D\cap \,{\varGamma }^{N} = \emptyset$.

The second problem is the two-dimensional plane strain elasticity problem which can be expressed in the following way

2 $$\begin{aligned}{}&{{\,\mathrm{div}\,}}\varvec{\sigma }+\textbf{b}=\textbf{0} \qquad \text {in } \varOmega \\&\textbf{u}=\hat{\textbf{u}} \quad \text {on } {\varGamma }^D\,, \qquad \varvec{\sigma }\cdot \textbf{n}=\hat{\textbf{t}} \quad \text {on } {\varGamma }^N\end{aligned}$$

where $\varvec{\sigma }$ is the stress tensor, $\textbf{b}$ is the body force vector, $\hat{\textbf{t}}$ is the prescribed traction force vector, $\textbf{u}$ is the displacement vector and $\hat{\textbf{u}}$ is the prescribed displacement vector.

Equation (2) is supplemented by Hooke’s law

3 $$\begin{aligned} \varvec{\sigma }= \textbf{E}: \varvec{\varepsilon }\qquad \text {in } \varOmega \end{aligned}$$

where $\varvec{\varepsilon }$ is the strain tensor, $\textbf{E}$ is the fourth order Hooke’s tensor, in which for the two-dimensional plane strain state the following non-zero terms are present

4 $$\begin{aligned} \begin{aligned}{}&(\textbf{E})_{1111} = (\textbf{E})_{2222} =\lambda + 2 \mu \,,\qquad (\textbf{E})_{1122} = (\textbf{E})_{2211} = \lambda \\&(\textbf{E})_{1212} = (\textbf{E})_{1221} = (\textbf{E})_{2112} = (\textbf{E})_{2121} = \mu \end{aligned} \end{aligned}$$

and $\lambda , \mu$ are standard elasticity constants.

Small strains are assumed in this paper, therefore the Cauchy strain tensor is used

5 $$\begin{aligned} \varvec{\varepsilon }= \frac{1}{2}\Bigl [{\varvec{\nabla }}\textbf{u}+ ({\varvec{\nabla }}\textbf{u})^{\mathrm{T}}\Bigr ] \quad \text {in } \varOmega \end{aligned}$$

**Figure 1:**
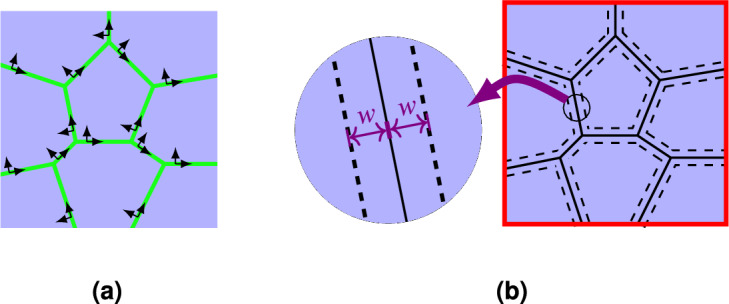
Mesh skeleton: local coordinates (**a**), interpretation of stability parameter $w$ (**b**).

In the DGFD method the discontinuities of the global approximation across the mesh skeleton ${\varGamma }^{d}$ need to be considered in the problem formulation. Local coordinates associated with the mesh skeleton are specified. They are based on two unit vectors $(\textbf{n}, {\textbf{s}})$, where $\textbf{n}$ is the unit vector normal to ${\varGamma }^{d}$ and ${\textbf{s}}$ is tangent to ${\varGamma }^{d}$. Fig. 1a presents the skeleton local coordinates, and Fig. 1b shows a graphical illustration of a stability parameter $w$. The parameter $w$ is related only to the mesh, not to the considered problem. Therefore, different types of boundary problems can be solved using the same mesh with a similar value of the stability parameter^63^. The numerical fluxes on the mesh skeleton are evaluated using finite difference formulas in the small neighborhood of the mesh skeleton which is defined by parameter $w$^3^. In the DGFD method, $w$ is not only a penalty-like parameter. It indicates points in the vicinity of the mesh skeleton, see Fig. 1b, where the trial function or its derivatives should be calculated to evaluate the numerical fluxes. It has been shown in other papers, for example^3–5,63^, that the DGFD method is stable and not sensitive to fluctuations of $w$. A wider discussion of parameter $w$ is presented in “Discussion on the stability parameter” Section.

In the discontinuous Galerkin method, the discontinuity and mean-value operators appear, which are defined with the help of the vector $\textbf{n}$ normal to the mesh skeleton. Their definitions for an auxiliary function $g$ are as follows:

6a $$\begin{aligned}{}&[ [ g ] ] _{\epsilon }\left(\textbf{x}\right) = g(\textbf{x}+ \epsilon \, {\textbf{n}^{d}_{}}) - g(\textbf{x}- \epsilon \, {\textbf{n}^{d}_{}})\\&[ [ g ] ] \left(\textbf{x}\right) = \lim _{\epsilon \rightarrow 0} [ [ g ] ] _{\epsilon }\left(\textbf{x}\right) &\text {for } \textbf{x}\in {\varGamma }^{d}\end{aligned}$$

6b $$\begin{aligned}{}&\left\langle g \right\rangle _{\epsilon }\left(\textbf{x}\right) = \frac{1}{2} \left( g(\textbf{x}+ \epsilon \, {\textbf{n}^{d}_{}}) + g(\textbf{x}- \epsilon \, {\textbf{n}^{d}_{}}) \right) \\&\left\langle g \right\rangle \left(\textbf{x}\right) = \lim _{\epsilon \rightarrow 0} \left\langle g \right\rangle _{\epsilon }\left(\textbf{x}\right) &\text {for } \textbf{x}\in {\varGamma }^{d}\end{aligned}$$

## Weak formulations

Following the procedures given in^2,5^ the problems presented in Eqs. (1) and (2) can be rewritten in weak forms. In both cases of the weak forms the test and trial functions belong to the same broken Sobolev space $S$, defined as follows

7 $$\begin{aligned} S= H^1(\varOmega ,\varOmega _h) = \left\{ g\in L^2(\varOmega ):\, g\Bigl |_{\varOmega ^e} \in H^1(\varOmega ^e)\, \forall \, \varOmega ^e \in \varOmega _h \right\} \end{aligned}$$

where $\varOmega _h$ represents the set of disjoint finite elements covering the whole domain $\varOmega$, and $\varOmega ^e$ is a single finite element. In the space $S$ a subset $\bar{S} \subset S$ can be distinguished that consists of functions which are continuous on the mesh skeleton and meet the boundary values on the Dirichlet boundary

8 $$\begin{aligned} \bar{S} = \left\{ g\in S: [ [ g ] ] =0 \text { on } {\varGamma }^{d}\,;\, g= \hat{u} \text { on } {\varGamma }^D\right\} \end{aligned}$$

In this section the weak form of Poisson’s problem is first presented, and then, avoiding certain repetitions, the weak form of the elasticity problem is shown. The weak form of Poisson’s problem in Eq. (1) is expressed as follows: Find $u\in \bar{S}$ such that

9 $$\begin{aligned} b({v},u) = l({v}) \qquad \forall \, {v}\in S\end{aligned}$$

where the bilinear and linear forms are defined as

10a $$b\left( {v,u} \right) = \int_{{}} {\nabla v \cdot \nabla ud\Omega + } \int\limits_{{\Gamma ^{d} }} {\left[\kern-0.15em\left[ v \right]\kern-0.15em\right]{\mathbf{n}} \cdot \nabla u{\text{d}}\Gamma - \int\limits_{{\Gamma ^{D} }} {v{\mathbf{n}} \cdot \nabla u{\text{d}}\Gamma } }$$

10b $$\begin{aligned} l({v})&=\int \limits _{\varOmega }{v}\, f\, \mathrm d\varOmega + \int \limits _{{\varGamma }^{N}}{v}\,\hat{h}\, \mathrm d{\varGamma }\end{aligned}$$

It is assumed in Eq. (9) that $\left\langle {\varvec{\nabla }}u \right\rangle \cdot \textbf{n}=0$ on ${\varGamma }^{d}$, which is a standard assumption in the computational methods such as FEM or other versions of the DG method. In this weak form, the test function belongs to the space of discontinuous functions, which is inconvenient when we want to find the approximate solution, since we want the trial function to belong to $\bar{S}$. Therefore, this formulation must be modified so that both the test and trial functions belong to the same space, however, the trial functions are forced to belong to set $\bar{S}$ when the stability parameter $w$ tends to zero. The modified weak form of Poisson’s problem is now expressed as follows:Find $u\in S$ such that:

11 $$\begin{aligned} b_w({v},u) =l({v}) + l_{w}({v}; \hat{u}) \qquad \forall \, {v}\in S\quad \text { and for } w\rightarrow 0 \end{aligned}$$

where

12a $$\begin{aligned} b_{w}({v},u)&= \int \limits _{\varOmega }{\varvec{\nabla }}{v}\cdot {\varvec{\nabla }}u\, \mathrm d\varOmega + \frac{3}{4} \int \limits _{{\varGamma }^{d}}\frac{1}{w}\, [ [ {v} ] ] [ [ u ] ] _{w} \, \mathrm d{\varGamma }- \frac{1}{2}\int \limits _{{\varGamma }^{d}} [ [ {v} ] ] \,\textbf{n}\cdot \left\langle {\varvec{\nabla }}u \right\rangle _{w} \, \mathrm d{\varGamma }\\&\quad +3\int \limits _{{\varGamma }^{D}}\frac{1}{w} {v}u(\textbf{x}_{2w}) \, \mathrm d{\varGamma }+4\int \limits _{{\varGamma }^{D}} {v}\textbf{n}\cdot {\varvec{\nabla }}u(\textbf{x}_{w})\, \mathrm d{\varGamma }+\int \limits _{{\varGamma }^{D}} {v}\textbf{n}\cdot {\varvec{\nabla }}u(\textbf{x}_{2w})\, \mathrm d{\varGamma }\end{aligned}$$

12b $$\begin{aligned}{} & l_{w}({v}; \hat{u})= \int \limits _{{\varGamma }^{D}}\frac{3}{w}\, {v}\hat{u} \, \mathrm d{\varGamma } \end{aligned}$$

where $\textbf{x}_w= \textbf{x}- w\, \textbf{n}$, $\textbf{x}_{2w} = \textbf{x}- 2\,w\, \textbf{n}$ and the value of the trial function at point $\textbf{x}_{w}$ on the Dirichlet boundary means the value of this function at distance $w$ from the boundary in the normal direction: $u(\textbf{x}_{w}) = u(\textbf{x}- \textbf{n}w)$ for $\textbf{x}\in {\varGamma }^D$. Now, we present the lemma showing that the formulations in Eqs. (9) and (11) are equivalent.

### Lemma 1

If there is a function $u\in S$ that satisfies Eq. (11), then the function belongs to ${\bar{S}}$ and satisfies Eq. (9).

### Proof

Equation (11) has to be satisfied for every value of $w$, and, in particular for an arbitrarily small value of the parameter. This means that every finite difference relation in this equation has to tend to a specific derivative for $w$ going to zero. Therefore, on the mesh skeleton we have

13 $$\frac{{\left[\kern-0.15em\left[ u \right]\kern-0.15em\right]_{w} }}{{2w}}\xrightarrow{{w \to 0}}{{\rm n}} \cdot \nabla u\;{\text{on}}\;\varGamma ^{d} \Rightarrow \left[\kern-0.15em\left[ u \right]\kern-0.15em\right] = 0\;{\rm on}\;\varGamma ^{d}$$

The finite difference on the mesh skeleton has to be finite for an arbitrarily small value of $w$, which indicates that the trial function has to be continuous on the mesh skeleton. On the Dirichlet boundary, we have the following finite difference relation

14 $$\frac{{\hat{u} - u({\mathbf{x}}_{w} )}}{{2w}}\xrightarrow{{w \to 0}}{{\rm n}} \cdot \nabla u\;{\text{on}}\;\varGamma ^{D} \Rightarrow u = \hat{u}\;{\text{on}}\;\varGamma ^{D}$$

In this case the finite difference on the Dirchlet boundary is also finite for every value of $w$, which indicates that the trial function has to meet the boundary values on the Dirichlet boundary. Additionally, it is obvious that the mean values of the derivative on the mesh skeleton tend to the derivative on the mesh skeleton, and a similar situation occurs with the derivative on the Dirichlet boundary

15 $$\begin{gathered} {\mathbf{n}} \cdot \left\langle {\nabla u} \right\rangle _{w} \xrightarrow{{w \to 0}}{\mathbf{n}} \cdot \nabla u\;{\text{on}}\;\varGamma ^{d} \hfill \\ {\mathbf{n}} \cdot \nabla u({\mathbf{x}}_{w} )\xrightarrow{{w \to 0}}{\mathbf{n}} \cdot \nabla u\;{\text{on}}\;\varGamma ^{D} \hfill \\ \end{gathered}$$

Equation (11) is now rewritten with some small modifications

16 $$\begin{aligned}{}&\int \limits _{\varOmega }{\varvec{\nabla }}{v}\cdot {\varvec{\nabla }}u\, \mathrm d\varOmega + \frac{3}{2} \int \limits _{{\varGamma }^{d}}\, [ [ {v} ] ] \frac{ [ [ u ] ] _{w}}{2w} \, \mathrm d{\varGamma }- \frac{1}{2}\int \limits _{{\varGamma }^{d}} [ [ {v} ] ] \,\textbf{n}\cdot \left\langle {\varvec{\nabla }}u \right\rangle _{w} \, \mathrm d{\varGamma }\\&\quad -6\int \limits _{{\varGamma }^{D}}{v}\, \frac{ \hat{u} - u(\textbf{x}_{2w})}{2 w} \, \mathrm d{\varGamma }+4\int \limits _{{\varGamma }^{D}} {v}\, \textbf{n}\cdot {\varvec{\nabla }}u(\textbf{x}_{w})\, \mathrm d{\varGamma }+\int \limits _{{\varGamma }^{D}} {v}\, \textbf{n}\cdot {\varvec{\nabla }}u(\textbf{x}_{2w})\, \mathrm d{\varGamma }= \int \limits _{\varOmega }{v}\, f\, \mathrm d\varOmega + \int \limits _{{\varGamma }^{N}}{v}\,\hat{h}\, \mathrm d{\varGamma }\end{aligned}$$

When $w$ goes to zero, then the finite difference relations in the second and fourth integrals in Eq. (16) tend to the derivatives as shown in Eqs. (13) and (14), and, using relations (15), this results in the following equation

17 $$\begin{aligned}{}&\int \limits _{\varOmega }{\varvec{\nabla }}{v}\cdot {\varvec{\nabla }}u\, \mathrm d\varOmega + \frac{3}{2} \int \limits _{{\varGamma }^{d}}\, [ [ {v} ] ] \, \textbf{n}\cdot {\varvec{\nabla }}u\, \mathrm d{\varGamma }- \frac{1}{2}\int \limits _{{\varGamma }^{d}} [ [ {v} ] ] \,\textbf{n}\cdot {\varvec{\nabla }}u\, \mathrm d{\varGamma }\\&\quad -6\int \limits _{{\varGamma }^{D}}{v}\, \textbf{n}\cdot {\varvec{\nabla }}u\, \mathrm d{\varGamma }+4\int \limits _{{\varGamma }^{D}} {v}\, \textbf{n}\cdot {\varvec{\nabla }}u\, \mathrm d{\varGamma }+\int \limits _{{\varGamma }^{D}} {v}\, \textbf{n}\cdot {\varvec{\nabla }}u\, \mathrm d{\varGamma }= \int \limits _{\varOmega }{v}\, f\, \mathrm d\varOmega + \int \limits _{{\varGamma }^{N}}{v}\,\hat{h}\, \mathrm d{\varGamma } \end{aligned}$$

in which $u\in \bar{S}$. After a few easy algebraic operations in Eq. (17), the Eq. (9) is retrieved. $\square$

To obtain a numerical solution, Eq. (11) has to be rewritten in a discrete version, in which the infinite functional space $S$ is substituted by the finite space $S^p \subset S$. The subspace $S^p$ comprises a complete set of polynomial basis functions in each element up to order $p$. In computations parameter $w$ cannot be arbitrarily small, the parameter is just small in the numerical sense. The Poisson’s problem shown in Eq. (11) is expressed in the discrete form as follows:Find $u^p \in S^p$ in $\varOmega$ such that

18 $$\begin{aligned} b_w({v}^p,u^p) = l({v}^p) + l_{w}({v}^p; \hat{u}) \qquad \forall \, {v}^p \in S^p \text { and for small value of w } \end{aligned}$$

The boundary value problem of elasticity in Eq. (2) also has its discrete form obtained in the same way as for the Poisson’s problem. It was derived in^4,5^ and reads Find $\textbf{u}^p \in S^p$ in $\varOmega$ such that

19 $$\begin{aligned} b^v_{w}(\textbf{v}^p,\textbf{u}^p) = l^v(\textbf{v}^p) + l^v_{w}(\textbf{v}^p; \hat{\textbf{u}}) \qquad \forall \, \textbf{v}^p \in S^p \quad \text { and for small value of } w\end{aligned}$$

The elasticity problem is a vector problem, so the definitions of the bilinear and linear forms are more complex:

20 $$\begin{aligned} b^v_{w}(\textbf{v},\textbf{u}) =&\int \limits _{{\varGamma }^{D}} \textbf{v}\cdot \textbf{E}^b_1\cdot \textbf{u}\, \mathrm d{\varGamma }-\int \limits _{{\varGamma }^{D}} \textbf{v}\cdot \textbf{E}^b_2:{\varvec{\nabla }}\textbf{u}\, \mathrm d{\varGamma }+\int \limits _{{\varGamma }^{D}} \textbf{v}\cdot \textbf{E}^b_3:{\varvec{\nabla }}\textbf{u}(\textbf{x}_{w} ) \, \mathrm d{\varGamma }\\ {}&+\int \limits _{{\varGamma }^{D}} \textbf{v}\cdot \textbf{E}^b_4:{\varvec{\nabla }}\textbf{u}(\textbf{x}_{2w} ) \, \mathrm d{\varGamma }\\ {}&+ \int \limits _{{\varGamma }^{d}} [ [ \textbf{v} ] ] \cdot \textbf{E}^d_1 \cdot [ [ \textbf{u} ] ] _{w} \, \mathrm d{\varGamma }- \int \limits _{{\varGamma }^{d}} [ [ \textbf{v} ] ] \cdot \textbf{E}^d_2 : \left\langle {\varvec{\nabla }}\textbf{u} \right\rangle _{w} \, \mathrm d{\varGamma }\\ {}&+ \int \limits _{{\varGamma }^{d}} [ [ \textbf{v} ] ] \cdot \textbf{E}^d_3 : \left\langle {\varvec{\nabla }}\textbf{u} \right\rangle \, \mathrm d{\varGamma }+ \int \limits _{\varOmega }{\varvec{\nabla }}\textbf{v}: \textbf{E}: {\varvec{\nabla }}\textbf{u}\, \mathrm d\varOmega \end{aligned}$$

21 $$\begin{aligned}{}& l^v(\textbf{v}) = \int \limits _{\varOmega }\textbf{v}\cdot \textbf{b}\, \mathrm d\varOmega +\int \limits _{{\varGamma }^{N}} \textbf{v}\cdot \hat{\textbf{t}} \, \mathrm d{\varGamma }\,, \quad l^v_{w}(\textbf{v}; \hat{\textbf{u}}) = \int \limits _{{\varGamma }^{D}} \textbf{v}\cdot \textbf{E}^b_1 \cdot \hat{\textbf{u}} \, \mathrm d{\varGamma }\end{aligned}$$

The tensors $\textbf{E}^d_i$ and $\textbf{E}^b_i$, related to the mesh skeleton and outer boundary, respectively, are defined as follows

22a $$\begin{aligned}{}&\textbf{E}^d_1 = \frac{3}{4w}\Bigl ( (\lambda + 2\mu )\,{\textbf{I}}- \lambda \,\textbf{s}\otimes \textbf{s}\Bigr ) \end{aligned}$$

22b $$\begin{aligned}{}&\textbf{E}^d_2 = \frac{1}{2}\Bigl ( (\lambda + 2\mu )\,(\textbf{n}\otimes \textbf{n}\otimes \textbf{n}) + 2\mu \, (\textbf{s}\otimes \textbf{s}\otimes \textbf{n}) \Bigr ) \end{aligned}$$

22c $$\begin{aligned}{}&\textbf{E}^d_3 = \lambda \, (\textbf{n}\otimes {\textbf{I}}- \textbf{n}\otimes \textbf{n}\otimes \textbf{n}) + 2\mu \, (\textbf{s}\otimes \textbf{n}\otimes \textbf{s}) \end{aligned}$$

22d $$\begin{aligned}{}&\textbf{E}^b_1 = \frac{3}{w}\Bigl ((\lambda + 2\mu )\,{\textbf{I}}- \lambda \textbf{s}\otimes \textbf{s}\Bigr ) \end{aligned}$$

22e $$\begin{aligned}{}&\textbf{E}^b_2 = \lambda \, \left( \textbf{n}\otimes \textbf{s}\otimes \textbf{s}\right) + 2\mu \,\left( \textbf{s}\otimes \textbf{n}\otimes \textbf{s}\right) \end{aligned}$$

22f $$\begin{aligned}{}&\textbf{E}^b_3 = 4\Bigl ((\lambda + 2\mu )(\textbf{n}\otimes \textbf{n}\otimes \textbf{n}) +2\mu (\textbf{s}\otimes \textbf{s}\otimes \textbf{n}) \Bigr ) \end{aligned}$$

22g $$\begin{aligned}{}&\textbf{E}^b_4 =(\lambda + 2\mu )(\textbf{n}\otimes \textbf{n}\otimes \textbf{n}) +2\mu (\textbf{s}\otimes \textbf{s}\otimes \textbf{n}) \end{aligned}$$

## Approximation in DG method

In the DG methods, the basis functions in the elements can be chosen as any sort of polynomial basis functions. In particular, they can just be monomials, Chebyshev, Legendre, or any other sort of polynomials, provided that they are complete up to order $p$. In certain situations, the basis functions can be enriched by another sort of functions, see^3^. In this paper, the approximation basis is chosen to be the Chebyshev polynomials, which are constructed using the recursive form as follows^64^

23 $$\begin{aligned}{}&T_0(\xi ) = 1\,, \quad T_1(\xi ) = \xi \,,\quad T_{n+1}(\xi ) = 2\cdot \xi \cdot T_{n}(\xi ) - T_{n-1}(\xi ) \end{aligned}$$

Orthogonality is a well-known property of the Chebyshev polynomials. However, they are not purely orthogonal, but orthogonal with the weight$\frac{1}{\sqrt{1-\xi ^2}}$ in the interval $[-1,\,1]$. The Chebyshev polynomials are more convenient for approximation due to their recursive definition, making it possible to calculate very high order polynomials without significant truncation errors. The basis functions are constructed in each element using the local coordinates starting at the center of the element

24 $$\begin{aligned} x^e=\frac{x-x_m^e}{0.5\,h^e_{x}}\,,\quad y^e=\frac{y-y_m^e}{0.5\,h^e_{y}} \end{aligned}$$

where the point $(x_m^e, y_m^e)$ is the centre of gravity of the *e*-th element cell and $h^e_{x}$, $h^e_{y}$ are the characteristic dimensions of the *e*-th cell in the *x* and *y* directions, respectively. The 2D basis functions are derived from the cross product of 1D polynomials:

25 $$\begin{aligned} \begin{aligned}{}&p=1\,: \quad \textbf{b}^e=\textbf{b}_1^e=\begin{bmatrix} 1&T_1(x^e)&T_1(y^e) \end{bmatrix} \\&p=2\,: \quad \textbf{b}^e=\textbf{b}_2^e=\begin{bmatrix} \textbf{b}_1^e&T_1(x^e) T_1(y^e)&T_2(x^e)&T_2(y^e) \end{bmatrix} \\&p=3\,: \quad \textbf{b}^e=\textbf{b}_3^e=\begin{bmatrix} \textbf{b}_2^e&T_3(x^e)&T_2(x^e) T_1(y^e)&T_1(x^e) T_2(y^e)&T_3(y^e) \end{bmatrix} \end{aligned} \end{aligned}$$

and so on. The global approximation, for the entire domain $\varOmega$, is constructed with the help of the global approximation matrix ${\Psi }^p$ and the vector of degrees of freedom $\mathbf {\check{u}}$. The suffix *p* in the symbol of the approximation matrix means that the polynomials of order up to *p* are used in the elements to compute the matrix. For example, the approximations for the scalar field $u$ and the vector field $\textbf{u}$ are as follows

26 $$\begin{aligned} u^p = {\Psi }^p \mathbf {\check{u}}\qquad \text {in } \varOmega \quad \text {or} \quad \textbf{u}^p = {\Psi }^p \mathbf {\check{u}}\qquad \text {in } \varOmega \end{aligned}$$

The matrix ${\Psi }^p$ is a one-row matrix for the scalar function $u$ in the Poisson’s problem given in Eq. (1) and two-row matrix for the 2D elasticity problem in Eq. (2).

**Figure 2:**
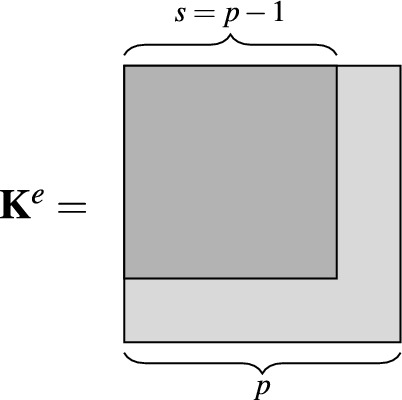
Hierarchical structure of element stiffness matrix of order *p*.

In the standard finite element method, the approximation is constructed with the help of the so-called shape functions, whose definitions depend on the shape of the finite element. In the DG methods, the basis functions do not depend on the shapes of the elements. The element matrices in the DG methods have the hierarchic structure as illustrated in Fig. 2. This means that the stiffness matrix of order $p$ keeps inside all the stiffness matrices of the lower orders. On the other hand, when we have the stiffness matrix of order $p$, it is relatively easy to extend it to the matrix of order $p+1$.

When the approximation in Eq. (26) is applied to the weak formulation of the problem in Eq. (11) or (18), we obtain the discrete problem in an algebraic form

27 $$\begin{aligned} \textbf{K}^p \mathbf {\check{u}}= \textbf{f}^p \end{aligned}$$

where $\textbf{K}^p$ and $\textbf{f}^p$ are defined depending on the problem under consideration

28a $$\begin{aligned}{}&\textbf{K}^p = b_w({\Psi }^p,{\Psi }^p) \,,\quad \textbf{f}^p = l({\Psi }^p) + l_{w}({\Psi }^p; \hat{u}) \end{aligned}$$

28b $$\begin{aligned}{}&\textbf{K}^p=b^v_{w}({\Psi }^p,{\Psi }^p) \,,\quad \textbf{f}^p = l^v({\Psi }^p) + l^v_{w}({\Psi }^p; \hat{\textbf{u}}) \end{aligned}$$

The DGFD method has been programmed in the Matlab environment which offers great flexibility and many up-to-date mathematical tools. Particularly, the subroutines for linear solvers are available in this environment. In this paper, the ’linsolve’ function has been applied for solving algebraic equations. The main problem in Eq. (27) is solved in the standard way.

## Discussion on the stability parameter

In Eqs. (18) and (19) the scalar stabilisation parameter $w$ plays an important role. It should cause the final discrete solution to be continuous and the Dirichlet boundary conditions to be met. The value of the parameter should be small enough for the compatibility conditions to be satisfied, but not too small to avoid numerical instabilities.

The $w$ parameter measures the small distance from the mesh skeleton, so its value depends on the size of the adjacent elements. On the other hand, its value is independent of the considered problem, but has to be adjusted to the mesh, so it is possible to use a single stabilization parameter for coupled problems^63^. The parameter cannot be arbitrarily small since numerical instabilities can occur if it is too small. In consequence, the solutions obtained this way are not strictly continuous and the Dirichlet boundary conditions are not exactly met. In other words, the value of the parameter should be chosen small enough to obtain good quality solutions, but not too small to avoid truncation errors that can spoil the solution.

The $w$ parameter should be small, however, it turns out that the final solution is not sensitive to its value. This means that a relatively large change in the value causes tiny changes in the approximate solution. This indicates that the DGFD method is consistent and well-defined. In the DGFD method, elements of various sizes and orders can be freely combined in the mesh, so the method is especially eligible for mesh adaptation. The stabilization parameter depends only on the mesh, i.e. its value depends only on the size of elements that adhere to the skeleton segment, $w= \gamma \cdot h$, where $h$ indicates the element size and $\gamma$ is a scaling parameter that is constant for the whole mesh skeleton.

**Figure 3:**
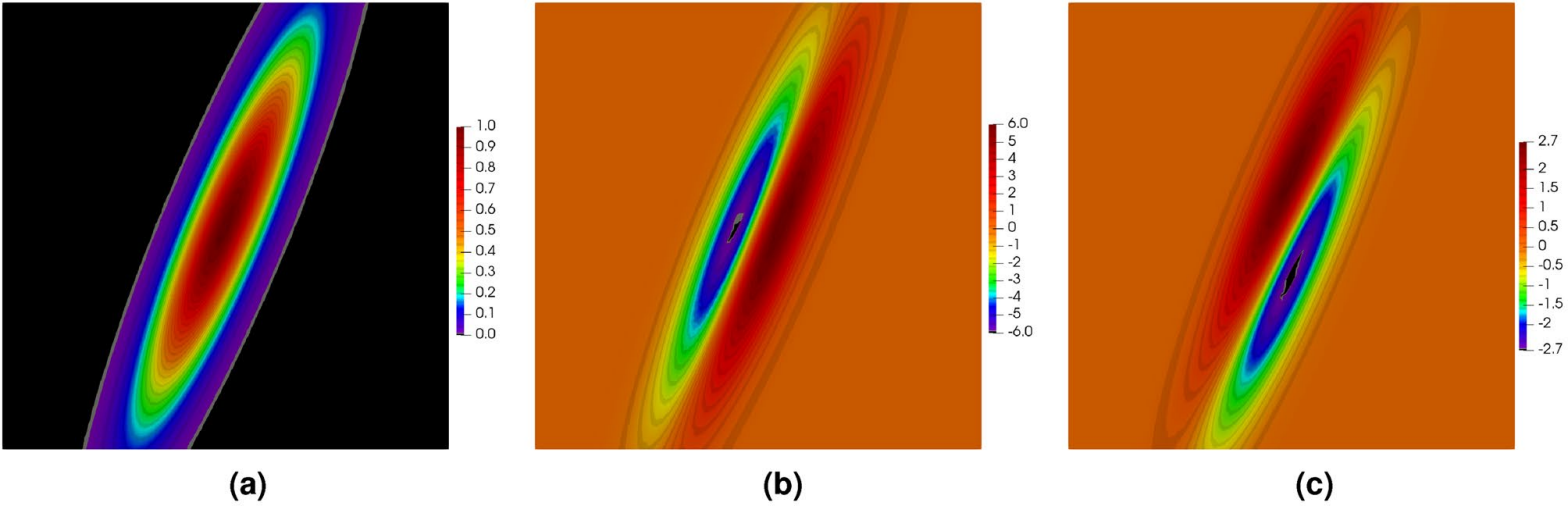
Function defined in Eq. (29) (**a**) and its derivatives in *x* direction (**b**) and *y* direction (**c**).

In order to illustrate the influence of the stabilization parameter on the final solution in the DGFD method, the Poisson’s problem, see Eq. (1), is solved in the square domain $\varOmega = [-1, 1] \times [-1, 1]$. The right-hand side function and the boundary conditions are taken from the exact solution, which is assumed to be the following exponential function

29 $$\begin{aligned}{}&u(x,y) = \exp \left( -\alpha \cdot \left( x^2 + (y-\beta x)^2 \right) \right) \end{aligned}$$

with the parameters $\alpha = 10$ and $\beta =2$. The function with its both derivatives is depicted in Fig. 3.

**Figure 4:**
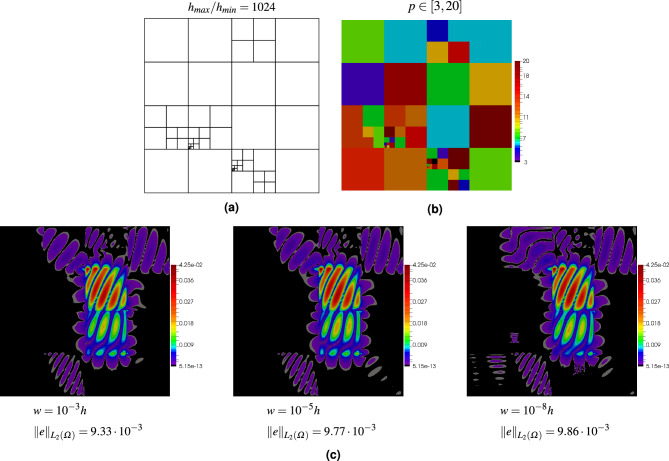
Numerical test of stability parameter *w* on the mesh with square elements: mesh structure (**a**), orders of elements (**b**), maps of errors for various values of $w$ (**c**).

**Figure 5:**
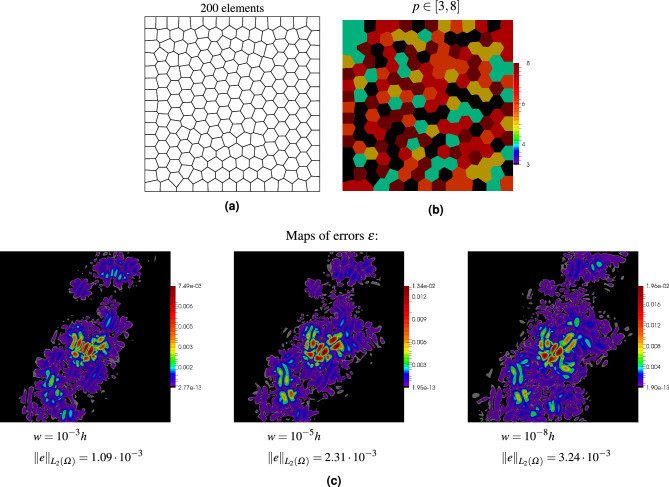
Numerical test of stability parameter *w* on the mesh with polygonal elements: mesh structure (**a**), orders of elements (**b**), maps of errors for various values of $w$ (**c**).

The problem has been solved with various values of scaling parameter $\gamma$ for quadrilateral and polygonal meshes. The meshes and maps of errors are shown in Figs. 4 and 5, respectively. In the quadrilateral mesh, the largest element is over one thousand times larger than the smallest element, and the two elements are adjacent to each other. The polynomial orders in the two meshes have been randomly generated: $p\in [3,20]$ for the quadrilateral mesh and $p\in [3,8]$ for the polygonal mesh. It can be seen in the two examples that even though the value of the stabilization parameter is changed by five orders of magnitude, only slight differences in the errors can be observed. Thus, the stability parameter in the DGFD method can be used to combine finite elements of different sizes as well as different orders.

## Error estimation method

In the proposed method of error approximation, the original boundary value problem is considered in a residuum form. The weak form of an error function is constructed, which is used to obtain an approximate error function. For the sake of clarity, the method is first presented for Poisson’s problem and then extended to the elasticity problem. The derivation begins from defining an exact error function as the difference between the exact solution $u$ and its approximation obtained by solving Eq. (18)

30 $$\begin{aligned} e= u- u^{p} \end{aligned}$$

The boundary value problem for the error function is constructed by applying the Laplace operator to both sides of Eq. (30) and substituting the right-hand side from Eq. (1)

31 $$\begin{aligned} -\Delta e= f- (-\Delta u^p ) \qquad \text {in } \varOmega \end{aligned}$$

Equation (31) must be completed by boundary conditions, which are as follows

32 $$\begin{aligned} e= \hat{e} = \hat{u} - u^p \qquad \text {on } {\varGamma }^D\,,\qquad {\varvec{\nabla }}e\cdot \textbf{n}= {\hat{h}}- {\varvec{\nabla }}u^p\cdot \textbf{n}\qquad \text {on } {\varGamma }^{N} \end{aligned}$$

It should be noted that $\hat{e}$ is not zero because in the DGFD method the Dirichlet boundary conditions are not strictly met. Thus, there are small differences between the prescribed values and the approximate solution on the ${\varGamma }^D$ boundary part, which are utilized to approximate the solution error. In the next step, both sides of Eq. (31) are converted into a weak form to obtain the following problem:For given function $u^p$ find function $e\in S$ such that

33 $$\begin{aligned} b_{w}({v},e) = l({v}) + l_{w}({v}; \hat{e}) - b({v},u^p) \qquad \forall \, {v}\in S\text { and for } w\rightarrow 0 \end{aligned}$$

The test as well as the trial functions in Eq. (33) belong to the infinite space, that is ${v},\, e\in S$, while the approximate solution belongs to the discrete space $u^p \in S^p$. We want to find an approximation of error $e$, and so the problem defined in Eq. (33) is rewritten in the finite space as follows:For given function $u^{p}\in S^p$ find approximation $e^{{\bar{p}}}\in S^{{\bar{p}}}$ such that

34 $$\begin{aligned} b_{w}({v}^{{\bar{p}}},e^{{\bar{p}}}) = l({v}^{{\bar{p}}}) + l_{w}({v}^{{\bar{p}}}; \hat{e}) - b({v}^{{\bar{p}}},u^p) \qquad \forall \, {v}^{{\bar{p}}} \in S^{{\bar{p}}} \text { and for small value of } w\end{aligned}$$

The symbol ${\bar{p}}$ indicates the order of approximation space, in which the error function is approximated. It is possible that ${\bar{p}}$ is the same as $p$, but much better results are obtained when ${\bar{p}}>p$. In this paper, it is assumed that ${\bar{p}}=p+1$. As it is noticed in “Approximation in DG method” Section, the enriched space in the DGFD method is quite easy to construct due to the hierarchical form of the basis functions.

A similar error approximation equation to the one presented in Eq. (34) can be generated for the elasticity problem. In that case the final problem of error approximation reads:For given $\textbf{u}^{p}$ find ${{\textbf {e}}}^{{\bar{p}}} \in S^{{\bar{p}}}$ in $\varOmega$ such that

35a $$\begin{aligned} b^v_{w}(\textbf{v}^{{\bar{p}}},{{\textbf {e}}}^{{\bar{p}}}) = l^v(\textbf{v}^p) + l^v_{w}(\textbf{v}^p; \hat{{{\textbf {e}}}}) - b^v(\textbf{v}^{{\bar{p}}},\textbf{u}^{p}) \qquad \forall \, \textbf{v}^p \in S^p \text { and for small value of }w\end{aligned}$$

where

35b $$\begin{aligned} b^v(\textbf{v},\textbf{u}) =&\int \limits _{\varOmega }{\varvec{\nabla }}\textbf{v}: \textbf{E}: {\varvec{\nabla }}\textbf{u}\, \mathrm d\varOmega + \int \limits _{{\varGamma }^{d}} [ [ \textbf{v} ] ] \otimes \textbf{n}: \textbf{E}: \left\langle {\varvec{\nabla }}\textbf{u} \right\rangle \, \mathrm d{\varGamma }- \int \limits _{{\varGamma }^{D}} \textbf{v}\otimes \textbf{n}: \textbf{E}: {\varvec{\nabla }}\textbf{u}\, \mathrm d{\varGamma }\end{aligned}$$

The error function $e^{{\bar{p}}}$ is approximated in the same manner as in Eq. (26), and using Eqs. (34) or (35a) the following system of algebraic equations is obtained

36 $$\begin{aligned}{}&e^{{\bar{p}}} = {\Psi }^{{\bar{p}}} \mathbf {\check{e}}\qquad \text {in } \varOmega \quad \rightarrow \quad \textbf{K}^{{\bar{p}}} \mathbf {\check{e}}= \textbf{r}^{{\bar{p}}} \end{aligned}$$

The approximation in the elements is constructed in the hierarchic way, as shown in “Approximation in DG method” Section and so, and therefore the system of algebraic equations in (36) is arranged in the following way

37 $$\begin{aligned} \textbf{K}^{{\bar{p}}}= \begin{bmatrix} \textbf{K}^{p} &{} \overline{\textbf{K}}\\ \overline{\textbf{K}}^{\mathrm{T}}&{} \overline{\overline{\textbf{K}}} \end{bmatrix} \,, \quad \mathbf {\check{e}}= \begin{bmatrix} \mathbf {\check{e}}_1\\ \mathbf {\check{e}}_2 \end{bmatrix} \,, \quad \textbf{r}^{{\bar{p}}}= \begin{bmatrix} \textbf{r}^{{\bar{p}}}_1 \\ \textbf{r}^{{\bar{p}}}_2 \end{bmatrix} \end{aligned}$$

The matrix $\textbf{K}^{p}$ has already been used to solve the Eq. (27), so it is already factorized. This is utilized in the error approximation

38a $$\begin{aligned}{}&\left( \overline{\overline{\textbf{K}}} - \overline{\textbf{K}}^{\mathrm{T}}{(\textbf{K}^{p})}^{-1}\overline{\textbf{K}}\right) \mathbf {\check{e}}_2 = \textbf{r}^{{\bar{p}}}_2 - \overline{\textbf{K}}^{\mathrm{T}}{(\textbf{K}^{p})}^{-1} \textbf{r}^{{\bar{p}}}_1 \end{aligned}$$

38b $$\begin{aligned}{}&\mathbf {\check{e}}_1 = {(\textbf{K}^{p})}^{-1} \textbf{r}^{{\bar{p}}}_1 - {(\textbf{K}^{p})}^{-1}\overline{\textbf{K}} \mathbf {\mathbf {\check{e}}}_2 \end{aligned}$$

The solution scheme shown in Eq. (38) requires the solution of the relatively small algebraic problem to get vector $\mathbf {\check{e}}_2$ and then vector $\mathbf {\check{e}}_1$ is obtained. The reduced system of algebraic Eq. (38a) is connected with the enriched part of the approximation space, which is much smaller in comparison to the main problem and therefore it is especially effective for high-order approximation.

## Examples

This section presents four benchmark examples, illustrating the performance of the developed error estimation method. Poisson’s problem is considered in the first two examples (“Exponential example for Poisson’s problem, Hyperbolic example for Poisson’s problem” Sections). The third example focuses on an elasticity problem (“Elasticity problem with singularity” Section). In the fourth example the Poisson’s problem is considered once again and an *hp* mesh adaptation is performed using the error estimation approach to identify the elements for the refinement. Error estimates are compared in each case with the exact error for various mesh densities and approximation orders. In order to present the quality of the error estimation the following efficiency index is defined for the approximate error in the whole domain and in a single finite element

39 $$\begin{aligned} \eta ^{{\bar{p}}} = \frac{\ln {\Vert e^{{\bar{p}}}\Vert _{L^2(\varOmega )}}}{\ln {\Vert e\Vert _{L^2(\varOmega )}}} \,,\qquad \eta ^{{\bar{p}}}_e = \frac{\ln {\Vert e^{{\bar{p}}}\Vert _{L^2(\varOmega ^e)}}}{\ln {\Vert e\Vert _{L^2(\varOmega ^e)}}} \end{aligned}$$

### Exponential example for Poisson’s problem

**Figure 6:**
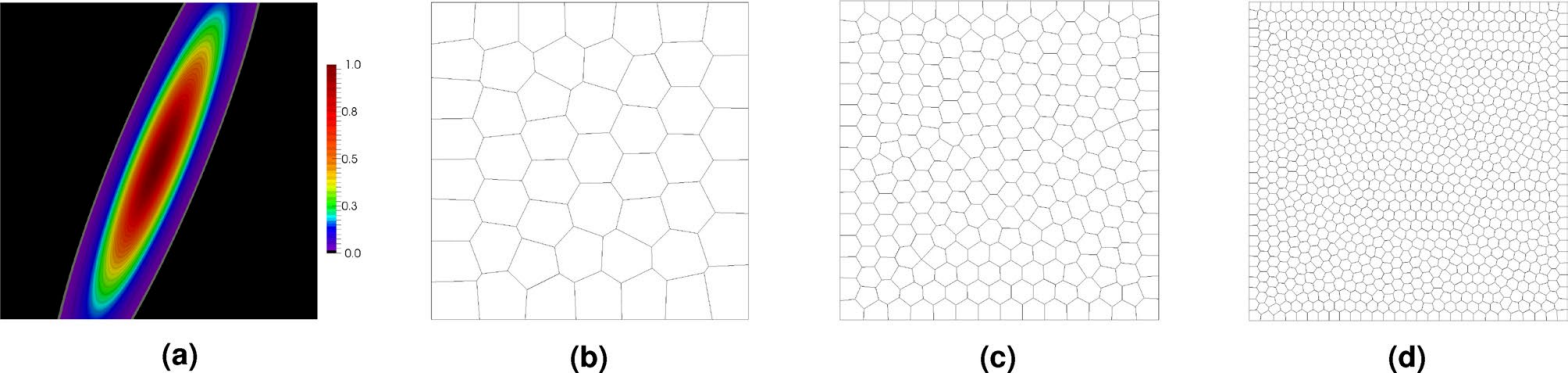
Example 1: exact solution (**a**), polygonal meshes with various numbers of elements: mp50 (**b**), mp250 (**c**) and mp1000 (**d**).

In this section, a benchmark Poisson’s problem is discussed whose exact solution is known a priori. It is the exponential function shown in Eq. (29) with $\alpha = 10$ and $\beta = 2$, depicted in Fig. 6a. It can be observed that the function is non-zero in the region close to the domain center, thus, the error concentrations are expected to appear there. Three polygonal meshes shown in Fig. 6b–d, comprising 50, 250, and 1000 elements, respectively, are used in this example. Additionally, quadrilateral and triangular meshes (not illustrated), consisting of 49/256/1024 and 50/242/1058 elements, respectively, are used in the calculations.

The calculations have been performed for three approximation orders $p=3,\,5,\,8$, for each type of mesh. The results are shown in Tables 1, 2 and 3 where the global error measure $\Vert e\Vert _{L^2(\varOmega )}$ is compared with the approximate global error $\Vert e^{{\bar{p}}}\Vert _{L^2(\varOmega )}$ with ${\bar{p}}=p+1$ and the efficiency indices are also provided in these tables. It is shown that the value of $\Vert e^{{\bar{p}}}\Vert _{L^2(\varOmega )}$  gets closer and closer to the exact value with the increase of mesh density and approximation order. It can be observed in Tables 1, 2 and 3 that the best results have been obtained for the polygonal meshes, while the least accurate results are for the triangular mesh. The rate of convergence for all kinds of meshes is the same, however, the error estimates for the triangular meshes show some irregularity, especially for higher order approximation. In the triangular elements, the vertex inner angles are acute, which may lead to some perturbations when the continuity of the approximate solution is enforced. It is the reason why the error estimation for the triangular elements does not show a stable performance, especially for high-order approximation. The polygonal finite elements, i.e. quadrilateral, pentagonal, and hexagonal elements, usually give better results in comparison to the triangular ones, which has been confirmed in this example.

**Table 1:** Example 1: exact and approximate global errors for polygonal meshes.

	$p$	$\Vert e\Vert _{L^2(\varOmega )}$	$\Vert e^{{\bar{p}}}\Vert _{L^2(\varOmega )}$	$\eta ^{{\bar{p}}}$
mp50	3	$7.09\times 10^{-2}$	$6.76\times 10^{-2}$	1.02
	5	$4.25\times 10^{-3}$	$4.06\times 10^{-3}$	1.01
	8	$7.98\times 10^{-5}$	$7.40\times 10^{-5}$	1.01
mp250	3	$3.48\times 10^{-3}$	$3.40\times 10^{-3}$	1.00
	5	$4.56\times 10^{-5}$	$4.31\times 10^{-5}$	1.01
	8	$9.83\times 10^{-8}$	$9.32\times 10^{-8}$	1.00
mp1000	3	$1.29\times 10^{-4}$	$1.23\times 10^{-4}$	1.01
	5	$7.45\times 10^{-7}$	$7.13\times 10^{-7}$	1.00
	8	$1.92\times 10^{-10}$	$1.83\times 10^{-10}$	1.00

**Table 2:** Example 1: exact and approximate global errors for quadrilateral meshes.

	$p$	$\Vert e\Vert _{L^2(\varOmega )}$	$\Vert e^{{\bar{p}}}\Vert _{L^2(\varOmega )}$	$\eta ^{{\bar{p}}}$
mqt49	3	$5.04\times 10^{-2}$	$4.48\times 10^{-2}$	1.04
	5	$7.38\times 10^{-3}$	$6.93\times 10^{-3}$	1.01
	8	$1.60\times 10^{-4}$	$1.42\times 10^{-4}$	1.01
mqt256	3	$4.22\times 10^{-3}$	$4.12\times 10^{-3}$	1.00
	5	$7.75\times 10^{-5}$	$7.53\times 10^{-5}$	1.00
	8	$1.94\times 10^{-7}$	$1.87\times 10^{-7}$	1.00
mqt1024	3	$3.84\times 10^{-4}$	$3.80\times 10^{-4}$	1.00
	5	$1.41\times 10^{-6}$	$1.35\times 10^{-6}$	1.00
	8	$4.20\times 10^{-10}$	$3.94\times 10^{-10}$	1.00

**Table 3:** Example 1: exact and approximate global errors for triangular meshes.

	$p$	$\Vert e\Vert _{L^2(\varOmega )}$	$\Vert e^{{\bar{p}}}\Vert _{L^2(\varOmega )}$	$\eta ^{{\bar{p}}}$
mtr50	3	$6.48\times 10^{-2}$	$5.66\times 10^{-2}$	1.05
	5	$1.45\times 10^{-2}$	$1.45\times 10^{-2}$	1.00
	8	$1.17\times 10^{-3}$	$5.73\times 10^{-4}$	1.11
mtr242	3	$4.59\times 10^{-3}$	$4.24\times 10^{-3}$	1.01
	5	$2.50\times 10^{-4}$	$2.40\times 10^{-4}$	1.00
	8	$1.98\times 10^{-6}$	$1.77\times 10^{-6}$	1.01
mtr1058	3	$2.72\times 10^{-4}$	$2.69\times 10^{-4}$	1.00
	5	$3.55\times 10^{-6}$	$3.52\times 10^{-6}$	1.00
	8	$1.34\times 10^{-9}$	$1.27\times 10^{-9}$	1.00

**Figure 7:**
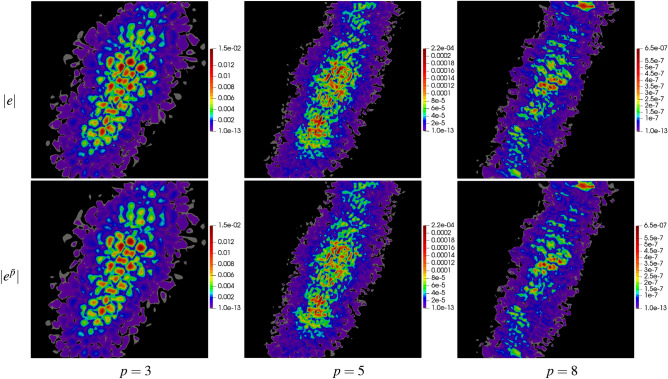
Example 1: maps of exact error $|e |$ and approximate error $|e^{{\bar{p}}} |$ on polygonal mesh with 250 elements for $p=3,\,5,\,8$.

**Figure 8:**
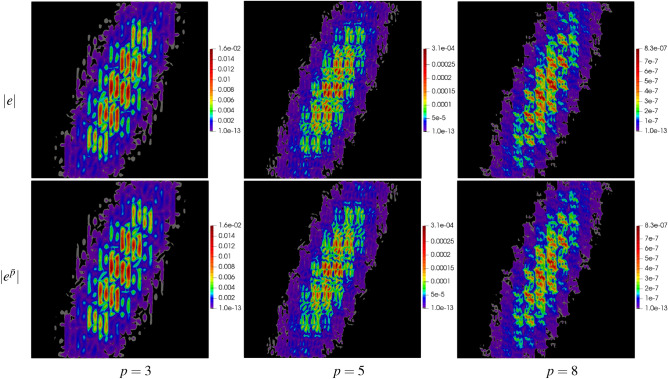
Example 1: maps of exact error $|e |$ and approximate error $|e^{{\bar{p}}} |$ on quadrilateral mesh with 256 elements for $p=3,\,5,\,8$.

**Figure 9:**
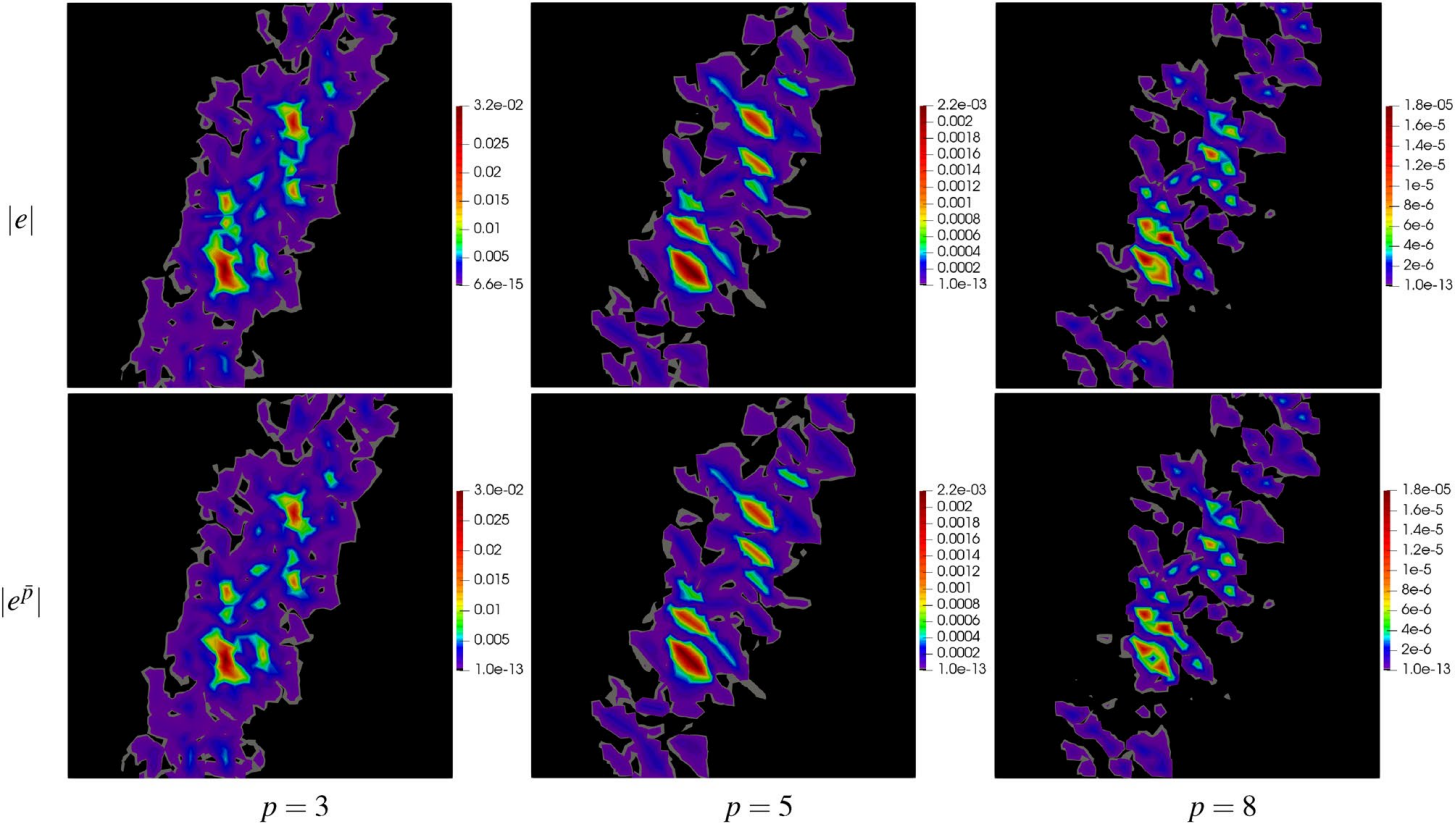
Example 1: maps of exact error $|e |$ and approximate error $|e^{{\bar{p}}} |$ on triangular mesh with 242 elements for $p=3,\,5,\,8$.

For a deeper analysis, the results have been presented in the form of error maps for three meshes: mp250, msq256, and mtr242 as well as for three values of $p=3,\,5,\,8$. The results are shown in Figs. 7, 8 and 9 in the form of maps of exact and approximate errors for the three meshes and the three approximation orders. As it is seen in the figures, the exact error is quite well recovered by the approximate error ${e^{{\bar{p}}}}$. In each case, the error distributions are reconstructed with a relatively high accuracy using the proposed method.

**Figure 10:**
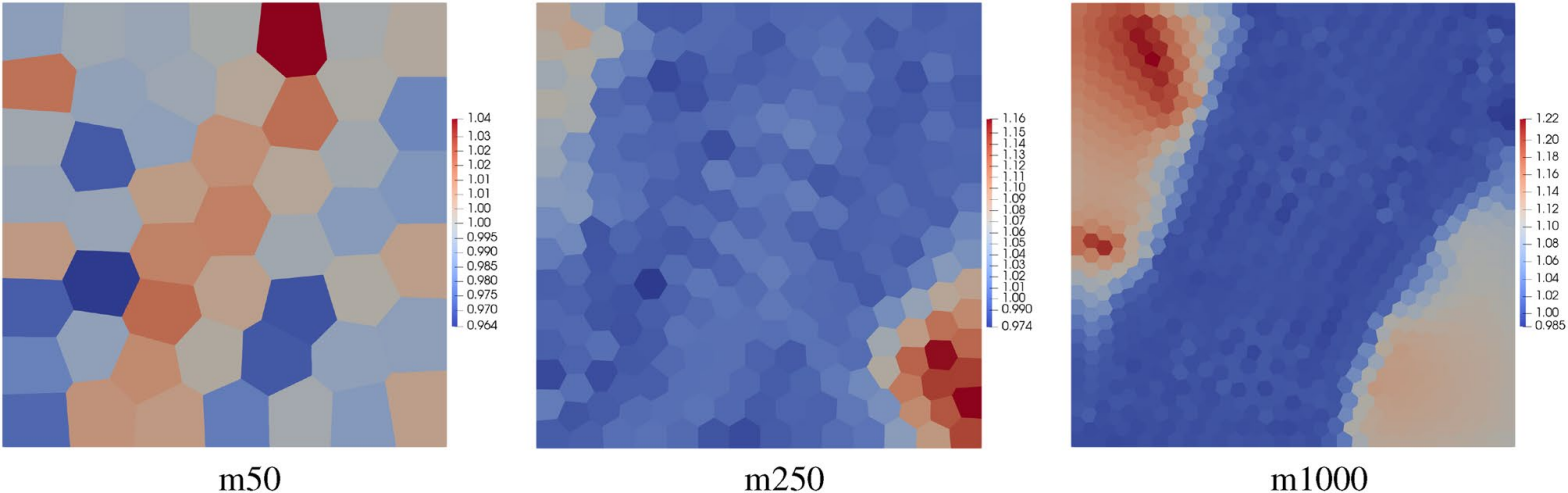
Example 1: maps of element error efficiency indexes for three meshes m50, m250 and m1000 with $p=5$.

Tab. 1 presents the global efficiency indexes for the polygonal meshes. For a deeper analysis of the error, the maps of the efficiency indexes over the fifth order elements for three meshes m50, m25 and m1000 are shown in Fig. 10. Comparing the maps of efficiency indexes with the error maps in Fig. 7 it can be concluded that the efficiency indexes are very close to one for the elements located at the places of error concentration. At the places where the error level is very small the element efficiency index tilts from unity. This means that the error approximation technique works well at the places with error concentration, however, at the places where the level of the error is very small its approximation may differ from the exact error values.

**Figure 11:**
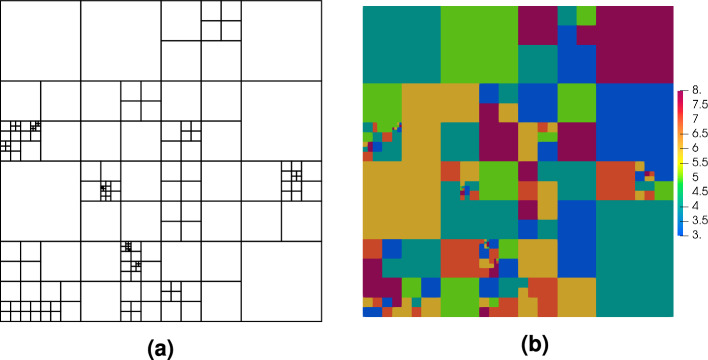
Example 1: randomly refined quadrilateral mesh (**a**) and map of randomly selected element orders (**b**).

**Figure 12:**
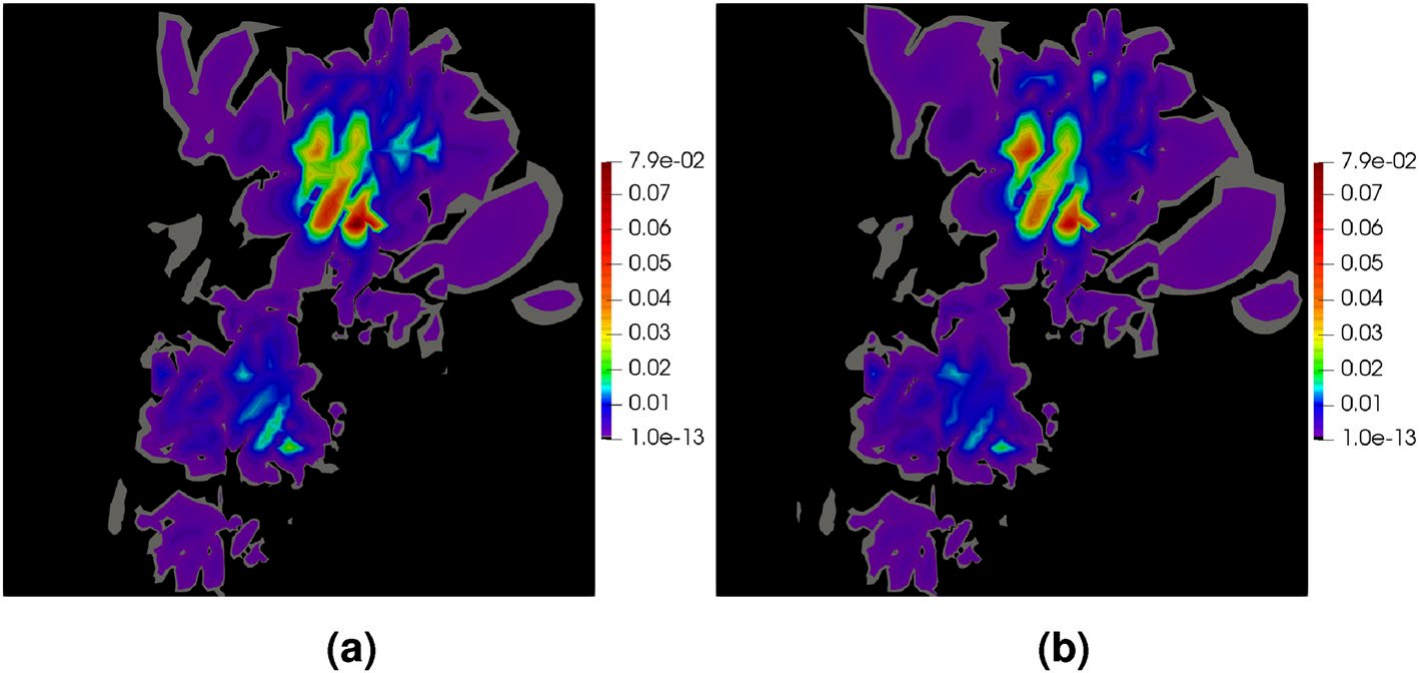
Example 1: map of exact error $|e |$ for the randomly generated quadrilateral mesh (**a**) and its approximation $|e^{{\bar{p}}} |$ (**b**).

So far, the error approximation methods discussed in this paper have been applied only to meshes with uniform approximation orders. However, the method is also suitable for unstructured meshes with non-uniform elements orders. The Poisson’s exponential benchmark has also been solved using a quadrilateral, randomly refined mesh where the orders of the elements have been chosen randomly from the range $p=2$ to $p=10$. The mesh structure and the map of the orders of the elements are depicted in Fig. 11. The analyzed error approximation technique is able to cope with such a strongly heterogeneous mesh because the error can be approximated with a relatively good accuracy as shown in Fig. 12.

### Hyperbolic example for Poisson’s problem

The Poisson’s problem defined in the square domain $[-1,\, 1] \times [-1,\, 1]$ is also the focus of this section. This time the exact solution is a function which is hyperbolic in the *y* direction and trigonometrical in the *x* direction

40 $$\begin{aligned} u(x,y) = \tanh (50 y) + \sin (x) + 2 \end{aligned}$$

**Figure 13:**
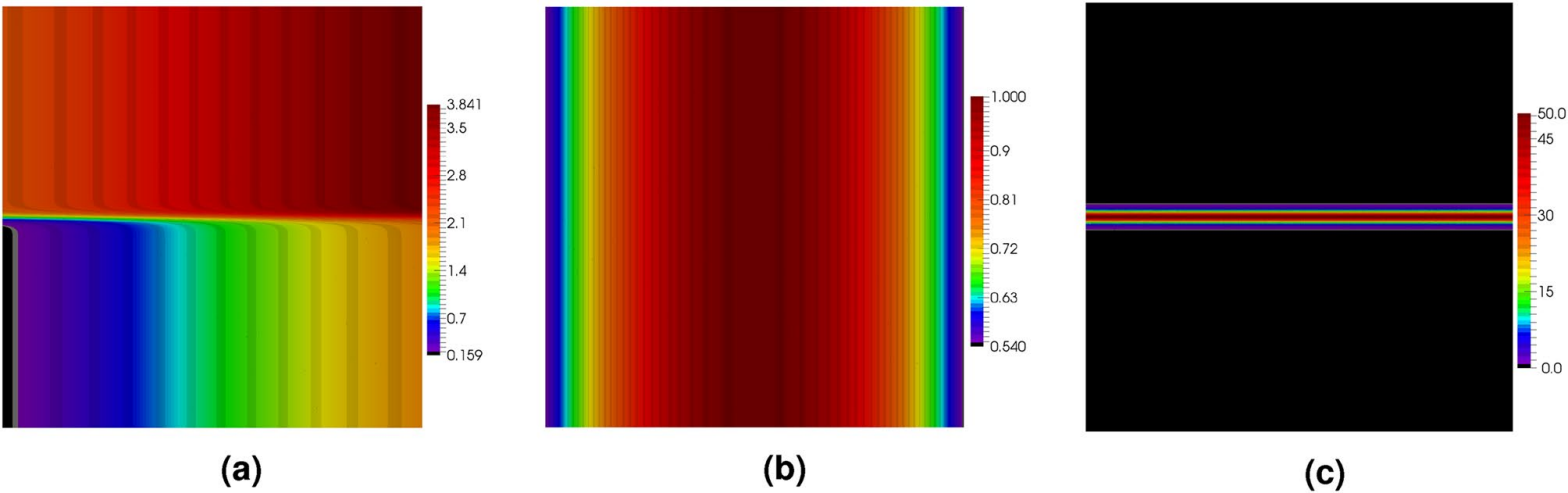
Example 2: exact solution $u$ (**a**) and its derivatives $u,_x$ (**b**) and $u,_y$ (**c**).

Figure 13 shows maps of the exact solution given in Eq. (40) and of its both derivatives. The function exhibits a strong change in the *y* direction for all points close to line $y=0$. The non-zero values of the derivative $u,_y$ appear only in the narrow band along the *x* axis, while the derivative $u,_x$ is quite smooth across the whole domain. Due to large gradients, errors are expected to occur in the regions close to line $y=0$.

**Figure 14:**
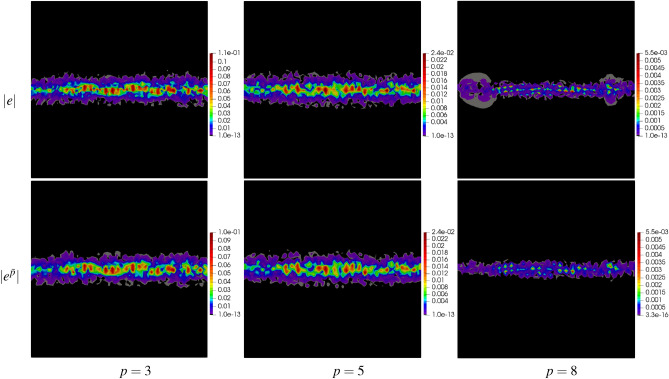
Example 2: maps of exact error $|e |$ and approximate error $|e^{{\bar{p}}} |$ on polygonal mesh with 1000 elements for $p=3,\,5,\,8$.

The domain has been discretized with the polygonal mesh mp1000, see Fig. 6d. Uniform approximation orders $p=3,\,5,\,8$ have been applied to all elements in the mesh. The global errors, their approximations and efficiency indices are shown in Table 4. The approximate global errors, once again, are very close to the exact errors. The maps of errors and their approximations are presented in Fig. 14, showing accurate concentrations of errors produced by the new error approximation technique.

**Table 4:** Example 2: exact and approximate global errors for polygonal meshes.

	$p$	$\Vert e\Vert _{L^2(\varOmega )}$	$\Vert e^{{\bar{p}}}\Vert _{L^2(\varOmega )}$	$\eta ^{{\bar{p}}}$
mp1000	3	$3.08\times 10^{-2}$	$2.66\times 10^{-2}$	1.04
	5	$4.27\times 10^{-3}$	$3.61\times 10^{-3}$	1.03
	8	$3.29\times 10^{-4}$	$2.69\times 10^{-4}$	1.02

### Elasticity problem with singularity

In this example, a benchmark elasticity problem, defined in Eq. (2), is analyzed. In the example^65^, the exact solution is written in terms of polar coordinates

41 $$\begin{aligned} \begin{aligned}{}&u_r(r,\theta ) = \frac{1}{2\mu } r^{\alpha } \left[ -(\alpha +1) \cos \left( (\alpha +1) \theta \right) + (C_2- \alpha -1 ) C_1 \cos \left( (\alpha -1) \theta \right) \right] \\ {}&u_\theta (r,\theta ) = \frac{1}{2\mu } r^{\alpha } \left[ (\alpha +1) \sin \left( (\alpha +1) \theta \right) + (C_2 +\alpha -1) C_1 \sin \left( (\alpha -1) \theta \right) \right] \end{aligned} \end{aligned}$$

where $C_1 = -\cos \left( (\alpha +1) \omega \right) /\cos \left( (\alpha -1) \omega \right)$, $C_2 = 2 \left( \lambda + 2\mu \right) / \left( \lambda + \mu \right)$, $\omega =3\pi /4$ and the critical exponent $\alpha$ is the positive solution of the equation $\alpha \sin (2\omega ) + \sin (2\omega \alpha ) = 0$, so that $\alpha \approx 0.544483737$.

The solution of Eq. (41) is singular at the origin since the stresses at this point tend to infinity. That is why in the approximate solution the error concentration is expected to appear around this point. The benchmark was originally solved on the so-called rotated L-shaped domain, see^65^. In this paper, the standard L-shaped domain with vertices at points: (− 1, − 1), (1, − 1), (1, 0), (0, 0), (0, 1), (− 1, 1) is considered. To obtain the solution in such a domain, both the global coordinates and the displacements need to be appropriately transformed, as shown below. The polar coordinates are constructed using Cartesian coordinates $\xi$ and $\eta$

42 $$\begin{aligned}{}[\theta ,r] = \text {cart2pol}(\xi ,\eta ) \end{aligned}$$

where the auxiliary coordinates $(\xi ,\eta )$ come from the following transformation of the global coordinates

43 $$\begin{aligned} \begin{aligned}{}&\phi =-3\pi /4 \\ {}&\xi = \cos (\phi ) {x} + \sin (\phi ) {y} \\ {}&\eta = -\sin (\phi ) {x} + \cos (\phi ) {y} \end{aligned} \end{aligned}$$

When the displacements are calculated in the polar coordinates, the displacements in the global coordinates are obtained with the same transformation angle $\phi$

44 $$\begin{aligned} \begin{aligned}{}&u_x(x,y) = \cos (\phi ) u_r - \sin (\phi ) u_\theta \\ {}&u_y(x,y) = \sin (\phi ) u_r + \cos (\phi ) u_\theta \end{aligned} \end{aligned}$$

**Figure 15:**
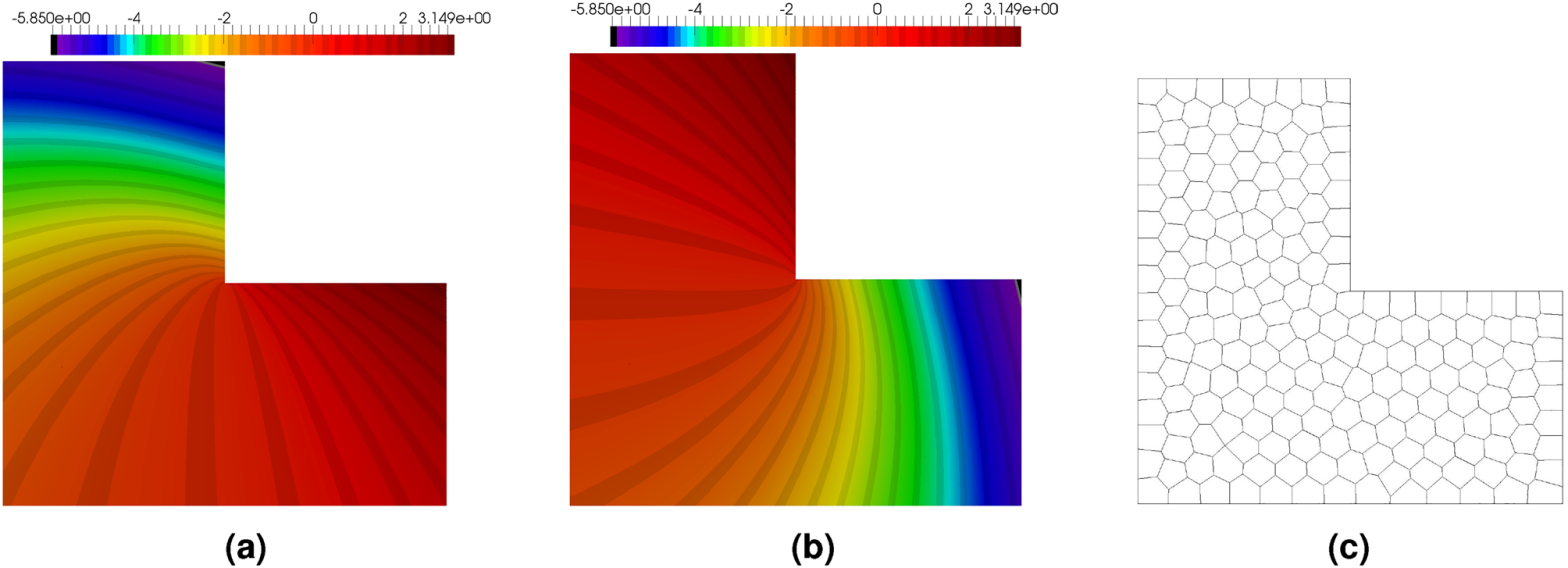
Example 3: component $u_x$ of exact solution (**a**), component $u_y$ of exact solution (**b**), polygonal mesh with 200 elements, lm200 (**c**).

Fig. 15 presents the maps of the exact solution of the considered problem as well as the polygonal mesh comprising 200 elements. The calculations have been performed for Young’s modulus $E= 1$ and $\nu =0.3$. The unified approximation order for all the elements has been chosen as $p=5$.

**Figure 16:**
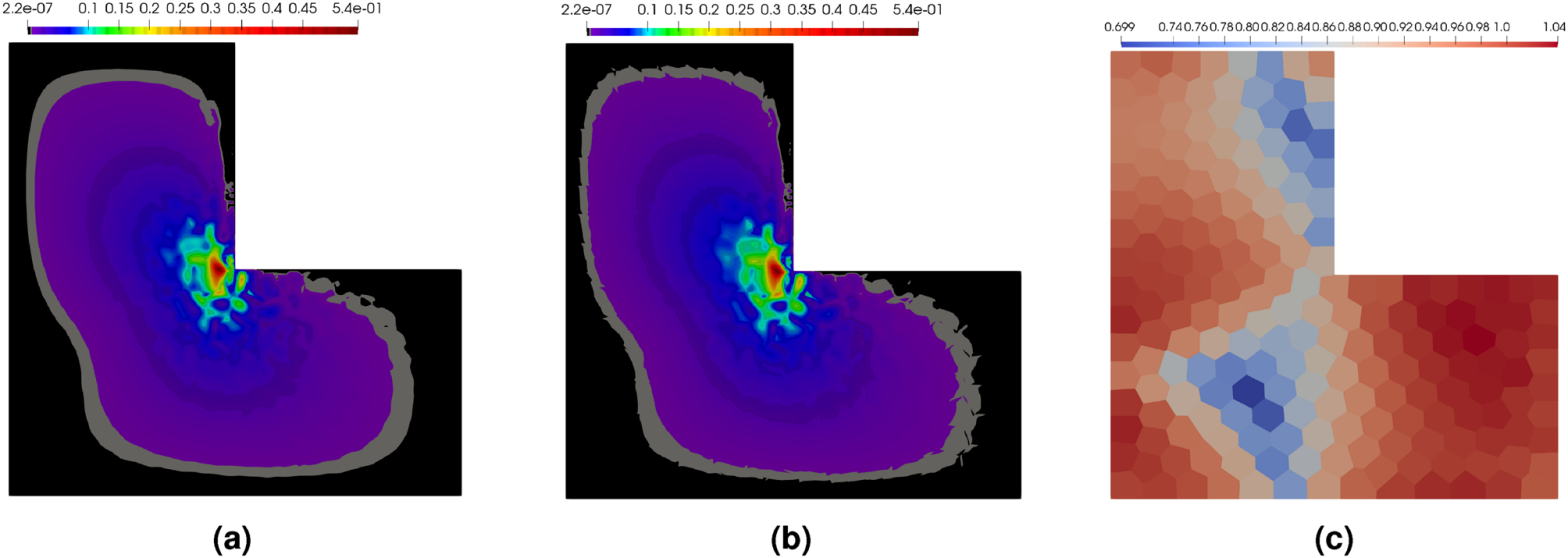
Example 3: exact error map $|{{\textbf {e}}} |$ (**a**), the error approximation $|{{\textbf {e}}}^{{\bar{p}}} |$ (**b**), and the map of the efficiency indexes in elements (**c**).

The maps of errors for the polygonal mesh, provided in Fig. 16a,b, enable the comparison of the exact and approximated errors. In this example, the error is concentrated, as expected, in the vicinity of the origin. The error approximation procedure is able to locate the place with the error concentrations and correctly recover the level of the error. The global efficiency index in this example is $\eta ^{{\bar{p}}}=0.5$, which can be caused by the error concentration in the very narrow region in the vicinity of the origin. For a deeper analysis of the situation the maps of the element efficiency indexes are presented in Fig. 16c. It can be noticed that in a large part of the domain the element efficiency indexes are close to one in most of the elements except those located in two regions where the indexes are about 0.7, however, the regions are not located near the origin. This example confirms that the procedure for error approximation performs well also for the elasticity problem.

### Poisson’s problem with hp mesh adaptation

**Figure 17:**
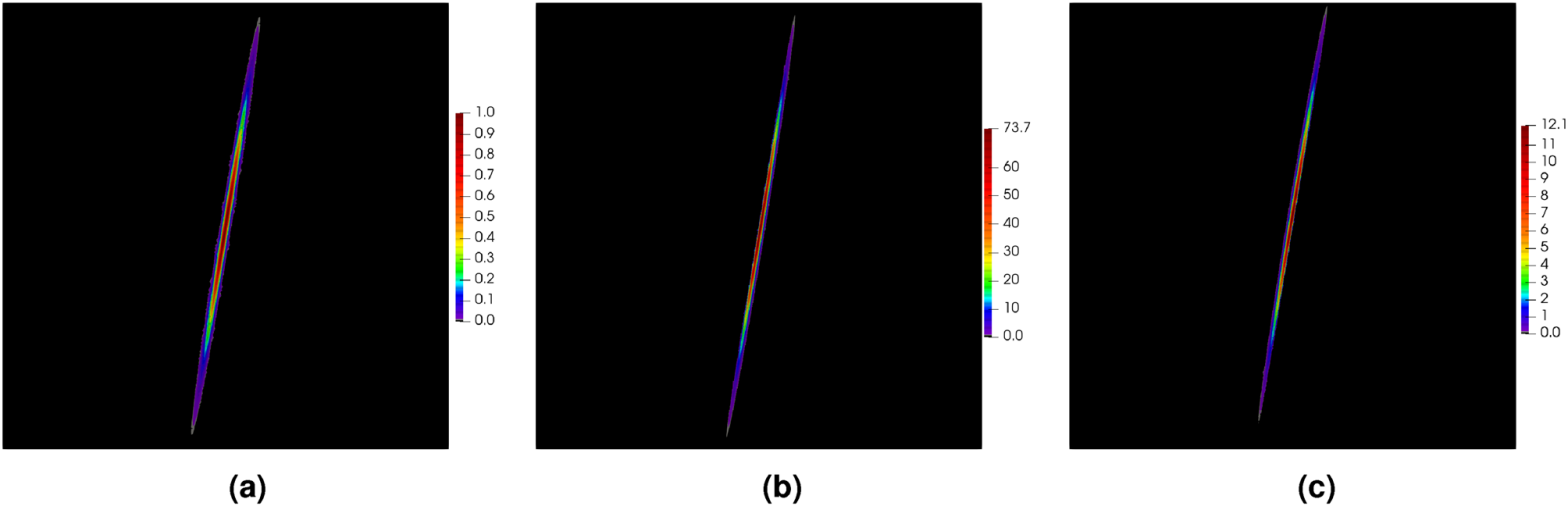
Example 4: exact solution $u(x,y)$ of Poisson’s problem in domain $[-1,\, 1] \times [-1,\, 1]$ (**a**), its derivatives in *x* direction (**b**) and in *y* direction (**c**).

In this example, we solve the benchmark Poisson’s problem whose exact solution in the domain $[-1,\, 1] \times [-1,\, 1]$ is the same as shown in Eq. (29), but now the values of the parameters are $\alpha = 200$ and $\beta = 6$. This causes the value of function $u(x,y)$ to be almost zero in the whole domain except the vicinity of a narrow band going through the domain, see Fig. 17.

In order to obtain an accurate solution by means of the discrete method the mesh should be refined along the narrow band. In this example, an automatic *hp* mesh refinement is applied using the a posteriori error approximation presented in this paper. In this example quadrilateral elements are used, since they enable flexible refinement in the DGFD method, as shown in the example in “Exponential example for Poisson’s problem” Section. In each step of the refinement, the problem is solved on the current mesh with the error approximation. Then, the error level for each element is calculated.

Various scenarios for the combined *h* and *p* mesh refinement can be applied. The main role of the automatic mesh adaptation is to minimize the global error and make the error uniformly distributed in the domain. The mesh adaptation is performed in refinement steps, in which the error is estimated after the problem solution on the current mesh. It is important to prepare a proper refinement scenario to reach the final mesh with a relatively small number of refinement steps. In this application the error is estimated for every finite element, then the elements with maximum and minimum errors are identified. In every adaptation step, two error parameters are set: the first is the 0.2 and 0.8 combination of the maximum and minimum errors in elements, respectively, and the second is the 0.05 and 0.95 combination of the same errors. The two error parameters are applied in the *hp* mesh refinement. The finite elements in which the error is higher than the second error parameter are *p* refined by increasing the approximation order by two and they are divided into four elements. Moreover, their neighbors are *p* refined by one and *h* refined by dividing them into four elements. The unrefined elements with errors greater than the first error parameter are divided into four elements. The whole procedure starts with a very coarse mesh, $3\times 3$ with second-order finite elements. In the first three steps, only the *h* refinement is applied to locate the places with higher errors. Afterward, the combined *hp* refinement is applied as described above. Many *hp* schemes can be applied to reach a similar final refined mesh. However, in this case, the *p* and *h* refinement are applied in every step of the refinement procedure to limit the number of iterations. Using this approach we obtained the final refined mesh in relatively few steps. It should be emphasized that we got the refined mesh that consists of elements with a wide spectrum of orders, from $p=2$ up to $p=13$. In the refinement procedure, the large and small finite elements as well as the elements with low and high order of approximation can be neighbors. It shows that the properties of the DGFD method allow for very flexible mesh refinement. The refinement of the *p* or *h* type can be applied only to a single element in the DGFD method without the need to interfere with the neighboring elements. In this example, the ability of the DGFD method to perform effectively combined mesh refinement is presented, and automated adaptivity is based on the effective error estimation in the elements.

**Figure 18:**
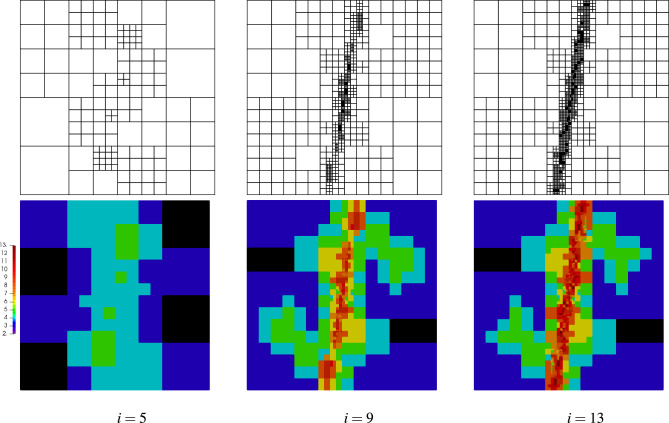
Example 4: meshes with maps of elements orders in selected steps of automatic *hp*–refinement.

**Figure 19:**
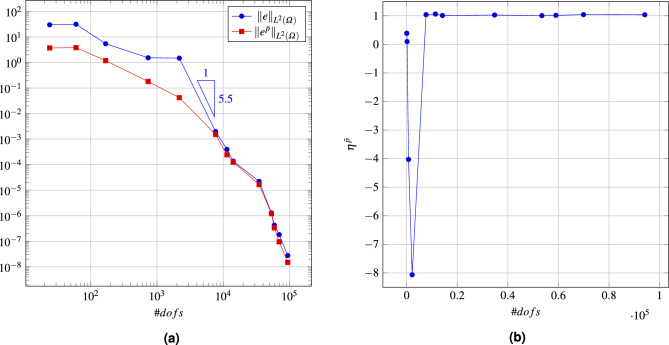
Example 4: convergence diagram in *hp* mesh refinement procedure (**a**), the efficiency indexes calculated in the refinement procedure (**b**).

**Figure 20:**
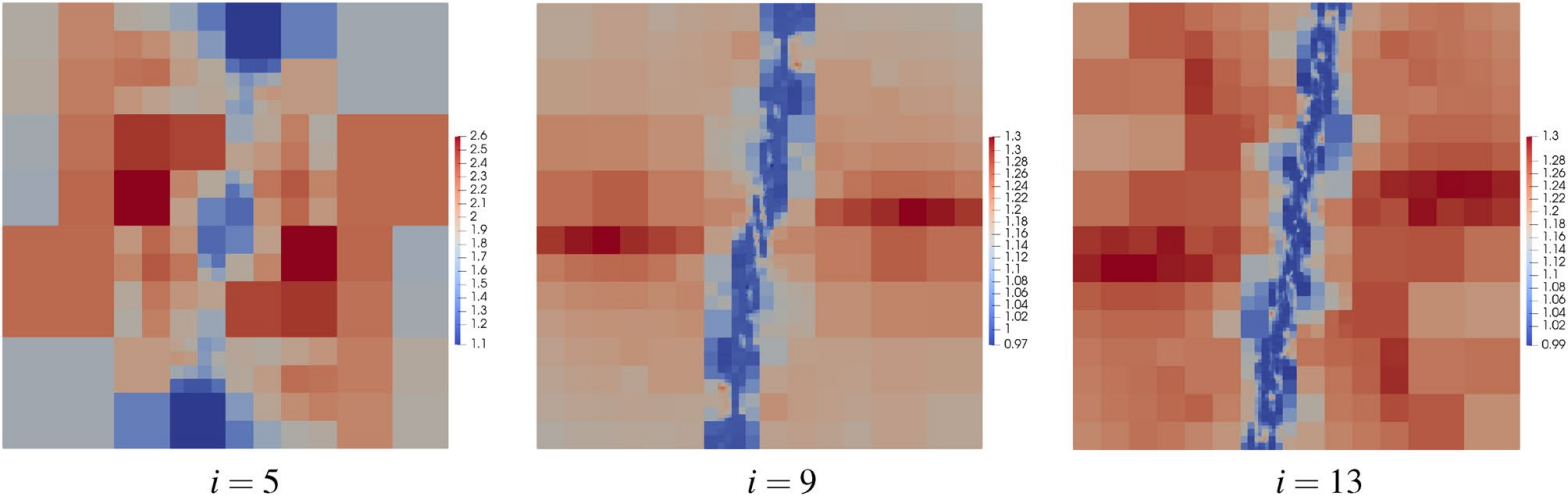
Example 4: maps of element efficiency indexes in selected steps of automatic *hp*–refinement.

Selected meshes obtained during the refinement procedure are shown in Fig. 18. It is seen that the mesh is mainly refined along the band where the errors are concentrated, so that the global error can be reduced with a relatively small number of degrees of freedom ($\#dofs$). In the first stage of the process, only the *h*-refinement is performed in order to identify the places with error concentration. In Fig. 19a the convergence diagram for the refinement is depicted. This is visible in the quite flat first part of the convergence diagram. After this stage, further refinement is practically concentrated in the vicinity of the specific band, which results in the steep second part of the diagram. The value of the efficiency index $\eta ^{{\bar{p}}}$ during the refinement procedure is shown in Fig. 19b. For the first five steps of the refinement the efficiency index is underestimated, however in the further steps of the refinement procedure the index values are close to one. The initial perturbations in the efficiency index are caused by the coarse mesh structure and the fact that the error is concentrated in the narrow band in the domain. For the refined mesh the value of the global error is correctly estimated. In the fifth step the efficiency index is about -8, where the real error was $1.5\times 10^{0}$ and the approximated error was $4.2\times 10^{-2}$. In Fig. 20 the maps of the element efficiency indexes are presented for the fifth, ninth and thirteenth adaptation steps. It can be noticed that in each case the indexes are close to one near the band with error concentration and the element indexes with higher values than one are generally at places with small error values.

## Conclusions

The paper presents the method for error approximation in the DGFD method for elliptic problems analyzed with various polygonal meshes. Some unique properties of the DGFD method are utilized to obtain an effective error approximation in the approximate solution. As a result, a new technique for error approximation is proposed.

Chebyshev polynomials are applied as the basis functions in the DGFD method. Their definition is recursive, thus they are hierarchical, which enables an efficient generation of the matrices for higher-order approximation space. The hierarchical formation of the stiffness matrix is utilized to get the error approximation developed in the upgraded approximation space.

The stability parameter *w* plays an important role in the DGFD method, since it is used for evaluating the so-called numerical fluxes on the mesh skeleton and for enforcing the continuity of the final solution. Generally, the stability parameter should be small for the compatibility of the final solution. However, the DGFD method is not very sensitive to the variations of this parameter, i.e. it can be changed significantly with a very small influence on the final solution. This property is utilized in this paper for error approximation techniques.

The method of error approximation has been illustrated with four benchmark examples, in which two kinds of two-dimensional elliptic problems, Poisson’s and elasticity problem, have been analyzed. It has been shown that the error approximation method on the polygonal meshes is able to locate places with error concentrations. In the last example, the method has been applied with success for an automatic *hp* mesh refinement. The method of error approximation can recover the exact error measure of high quality for polygonal meshes, including quadrilateral and triangular ones.

So far the error approximation method for the DGFD method has been applied in 2D elliptic problems. In the further research the method will be applied to other problems like linear and non-linear Navier–Stokes problem, or diffusion-convection transport in a compressible or incompressible medium.

## Data Availability

The datasets used and/or analysed during the current study available from the corresponding author on reasonable request.

## References

[1] Jaśkowiec J (2015). Discontinuous Galerkin method on reference domain. Comput. Assist. Meth. Eng. Sci..

[2] Jaśkowiec J (2017). The discontinuous Galerkin method with higher degree finite difference compatibility conditions and arbitrary local and global basis functions. Comput. Assist. Meth. Eng. Sci..

[3] Jaśkowiec J, Pluciński P, Stankiewicz A (2016). Discontinuous Galerkin method with arbitrary polygonal finite elements. Fin. Elem. Anal. Des..

[4] Jaśkowiec J (2017). Very high-order discontinuous Galerkin method in elliptic problems. Comput. Mech..

[5] Jaśkowiec J (2017). Application of discontinuous Galerkin method to mechanical 2D problem with arbitrary polygonal and high-order finite elements. Comput. Meth. Appl. Mech. Eng..

[6] Reed W, Hill T (1973). Triangular Mesh Methods for the Neutron Transport Equation. Report LA-UR-73-479.

[7] Cockburn, B. B., Karniadakis, G. & Shu, C.-W. (eds.) *Discontinuous Galerkin methods : theory, computation, and applications*. Lecture notes in computational science and engineering (Springer, 2000).

[8] Di Pietro D, Ern A (2011). Mathematical Aspects of Discontinuous Galerkin Methods. Mathématiques et Applications.

[9] Hesthaven JS, Warburton T (2007). Nodal Discontinuous Galerkin Methods: Algorithms, Analysis, and Applications.

[10] Mu L, Wang J, Wang Y, Ye X (2014). Interior penalty discontinuous Galerkin method on very general polygonal and polyhedral meshes. J. Comput. Appl. Math..

[11] Heimann F, Engwer C, Ippisch O, Bastian P (2013). An unfitted interior penalty discontinuous Galerkin method for incompressible Navier–Stokes two-phase flow. Int. J. Numer. Meth. Fluids.

[12] Rivière, B. *Discontinuous Galerkin Methods for Solving Elliptic and Parabolic Equations: Theory and Implementation*. Frontiers in Applied Mathematics (Society for Industrial and Applied Mathematics, 2008).

[13] Nitsche J (1971). Über ein variationsprinzip zur lösung von dirichlet-problemen bei verwendung von teilräumen, die keinen randbedingungen unterworfen sind. Abhandlungen aus dem Mathematischen Seminar der Universität Hamburg.

[14] Cockburn B, Shu C-W (1998). The local discontinuous Galerkin method for time-dependent convection-diffusion systems. SIAM J. Numer. Anal..

[15] Castillo P (2006). A review of the local Discontinuous Galerkin (LDG) method applied to elliptic problems. Appl. Numer. Math..

[16] Rupp A, Knabner P (2017). Convergence order estimates of the local discontinuous Galerkin method for instationary Darcy flow. Numer. Meth. Part. Differ. Equs..

[17] Bassi F, Rebay S (1997). A high-order accurate discontinuous finite element method for the numerical solution of the compressible Navier–Stokes equations. J. Comput. Phys..

[18] Baumann CE, Oden JT (1999). A discontinuous $$hp$$ finite element method for convection-diffusion problems. Comput. Meth. Appl. Mech. Eng..

[19] Cockburn B, Gopalakrishnan J (2004). A characterization of hybridized mixed methods for second order elliptic problems. SIAM J. Numer. Anal..

[20] Samii A, Panda N, Michoski C, Dawson C (2016). A hybridized discontinuous Galerkin method for the nonlinear Korteweg-de Vries equation. J. Sci. Comput..

[21] Cockburn B, Gopalakrishnan J, Lazarov R (2009). Unified hybridization of discontinuous Galerkin, mixed, and continuous Galerkin methods for second order elliptic problems. SIAM J. Numer. Anal..

[22] Nguyen NC, Peraire J, Cockburn B (2011). An implicit high-order hybridizable discontinuous Galerkin method for the incompressible Navier–Stokes equations. J. Comput. Phys..

[23] Zhao J, Zhao W, Xu Y (2023). Hybridizable discontinuous Galerkin methods for space-time fractional advection-dispersion equations. Appl. Math. Comput..

[24] Troescher N, Higdon J (2023). A fully-implicit hybridized discontinuous Galerkin spectral element method for two phase flow in petroleum reservoirs. J. Comput. Phy..

[25] Demkowicz LF, Gopalakrishnan J (2014). An Overview of the Discontinuous Petrov Galerkin Method.

[26] Keith B (2017). An ultraweak DPG method for viscoelastic fluids. J. Non-Newton. Fluid Mech..

[27] Borker R, Farhat C, Tezaur R (2017). A discontinuous Galerkin method with Lagrange multipliers for spatially-dependent advection–diffusion problems. Comput. Meth. Appl. Mech. Eng..

[28] Arnold DN, Brezzi F, Cockburn B, Marini LD (2002). Unified analysis of discontinuous Galerkin methods for elliptic problems. SIAM J. Numer. Anal..

[29] Kirby RM, Karniadakis GE (2005). Selecting the numerical flux in discontinuous Galerkin methods for diffusion problems. J. Sci. Comput..

[30] Segeth K (2010). A review of some a posteriori error estimates for adaptive finite element methods. Math. Comput. Simul..

[31] Zhang W, Nie Y, Gu Y (2015). Adaptive finite element analysis of elliptic problems based on bubble-type local mesh generation. J. Comput. Appl. Math..

[32] Zboinski G (2013). Adaptive $$hpq$$ finite element methods for the analysis of 3D-based models of complex structures. Part 2. A posteriori error estimation.. Comput. Meth. Appl. Mech. Eng..

[33] Kamenski L (2012). A study on using hierarchical basis error estimates in anisotropic mesh adaptation for the finite element method. Eng. Comput..

[34] Jaworska I, Orkisz J (2017). Estimation of a posteriori computational error by the higher order multipoint meshless FDM. Comput. Inform..

[35] Amani J, Bagherzadeh AS, Rabczuk T (2014). Error estimate and adaptive refinement in mixed discrete least squares meshless method. Math. Probl. Eng..

[36] Hartmann R, Houston P (2003). Adaptive discontinuous Galerkin finite element methods for nonlinear hyperbolic conservation laws. SIAM J. Sci. Comput..

[37] Krivodonova L, Flaherty JE (2003). Error estimation for discontinuous Galerkin solutions of two-dimensional hyperbolic problems. Adv. Comput. Math..

[38] Adjerid S, Baccouch M (2007). The discontinuous Galerkin method for two-dimensional hyperbolic problems. Part I: Superconvergence error analysis. J. Sci. Comput..

[39] Adjerid S, Baccouch M (2009). The discontinuous Galerkin method for two-dimensional hyperbolic problems Part II: A Posteriori error estimation. J. Sci. Comput..

[40] Adjerid S, Baccouch M (2010). Asymptotically exact a posteriori error estimates for a one-dimensional linear hyperbolic problem. Appl. Numer. Math..

[41] Adjerid S, Mechai I (2014). A superconvergent discontinuous Galerkin method for hyperbolic problems on tetrahedral meshes. J. Sci. Comput..

[42] Rivière B, Wheeler M (2003). A posteriori error estimates for a discontinuous Galerkin method applied to elliptic problems. Log number: R74. Comput. Math. Appl..

[43] Tabarraei A, Sukumar N (2007). Adaptive computations using material forces and residual-based error estimators on quadtree meshes. Comput. Meth. Appl. Mech. Eng..

[44] Houston P, Schötzau D, Wihler TP (2007). Energy norm a posteriori error estimation of $$hp$$-adaptive discontinuous Galerkin methods for elliptic problems. Math. Mod. Meth. Appl. Sci..

[45] Giani S, Grubisic L, Hakula H, Ovall JS (2018). An a posteriori estimator of eigenvalue/eigenvector error for penalty-type discontinuous Galerkin methods. Appl. Math. Comput..

[46] Hakula H, Neilan M, Ovall JS (2017). A posteriori estimates using auxiliary subspace techniques. J. Sci. Comput..

[47] Baccouch M (2018). Superconvergence of the local discontinuous Galerkin method for the sine-Gordon equation in one space dimension. J. Comput. Appl. Math..

[48] Baccouch M (2017). A superconvergent local discontinuous Galerkin method for nonlinear two-point boundary-value problems. Numer. Algorithms.

[49] Baccouch M (2017). A recovery-based error estimator for the discontinuous Galerkin method for transient linear hyperbolic conservation laws on Cartesian grids. Int. J. Comput. Methods.

[50] Baccouch M (2017). A posteriori error estimates and adaptivity for the discontinuous Galerkin solutions of nonlinear second-order initial-value problems. Appl. Numer. Math..

[51] Baccouch M (2017). A posteriori error estimator based on derivative recovery for the discontinuous Galerkin method for nonlinear hyperbolic conservation laws on Cartesian grids. Numer. Methods Part. Differ. Equs..

[52] Baccouch M (2017). Superconvergence of the discontinuous Galerkin method for nonlinear second-order initial-value problems for ordinary differential equations. Appl. Numer.l Math..

[53] Baccouch M (2018). A posteriori local discontinuous Galerkin error estimates for the one-dimensional sine-Gordon equation. Int. J. Comput. Math..

[54] Baccouch M (2017). Superconvergence of the local discontinuous Galerkin method for the sine-Gordon equation on Cartesian grids. Appl. Numer. Math..

[55] Baccouch M (2017). An optimal a posteriori error estimates of the local discontinuous Galerkin method for the second-order wave equation in one space dimension. Int. J. Numer. Anal. Model..

[56] Baccouch M (2016). A posteriori error analysis of the discontinuous Galerkin method for two-dimensional linear hyperbolic conservation laws on Cartesian grids. J. Sci. Comput..

[57] Baccouch M (2016). Optimal a posteriori error estimates of the local discontinuous Galerkin method for convection-diffusion problems in one space dimension. J. Comput. Math..

[58] Baccouch M (2016). Analysis of a posteriori error estimates of the discontinuous Galerkin method for nonlinear ordinary differential equations. Appl. Numer. Math..

[59] Baccouch M (2016). Recovery-based error estimator for the discontinuous Galerkin method for nonlinear scalar conservation laws in one space dimension. J. Sci. Comput..

[60] Baccouch M, Temimi H (2016). Analysis of optimal error estimates and superconvergence of the discontinuous Galerkin method for convection-diffusion problems in one space dimension. Int. J. Numer. Anal. Model..

[61] Baccouch M, Adjerid S (2011). Discontinuous Galerkin error estimation for hyperbolic problems on unstructured triangular meshes. Comput. Methods Appl. Mech. Eng..

[62] Cui J, Gao F, Sun Z, Zhu P (2020). A posteriori error estimate for discontinuous Galerkin finite element method on polytopal mesh. Numer. Methods Part. Differ. Equs..

[63] Jaśkowiec J, Pluciński P (2018). Discontinuous Galerkin method in numerical simulation of two-dimensional thermoelasticity problem with single stabilization parameter. Adv. Eng. Softw..

[64] Doman B (2015). The Classical Orthogonal Polynomials.

[65] Carstensen C, Gedicke J (2016). Robust residual-based a posteriori Arnold–Winther mixed finite element analysis in elasticity. Comput. Methods Appl. Mech. Eng..

